# In situ cell-type-specific cell-surface proteomic profiling in mice

**DOI:** 10.1016/j.neuron.2022.09.025

**Published:** 2022-10-10

**Authors:** S. Andrew Shuster, Jiefu Li, URee Chon, Miley C. Sinantha-Hu, David J. Luginbuhl, Namrata D. Udeshi, Dominique Kiki Carey, Yukari H. Takeo, Qijing Xie, Chuanyun Xu, D.R. Mani, Shuo Han, Alice Y. Ting, Steven A. Carr, Liqun Luo

**Affiliations:** 1 Department of Biology and Howard Hughes Medical Institute, Stanford University, Stanford, CA 94305, USA; 2 Neurosciences Program, Stanford University, CA 94305, USA; 3 The Broad Institute of MIT and Harvard, Cambridge, MA 02142, USA; 4 Departments of Genetics, Biology, and Chemistry, Chan Zuckerberg Biohub, Stanford University, Stanford, CA 94305, USA; 5 These authors contributed equally to this work.; 6 Lead Contact

## Abstract

Cell-surface proteins (CSPs) mediate intercellular communication throughout the lives of multicellular organisms. However, there are no generalizable methods for quantitative CSP profiling in specific cell types in vertebrate tissues. Here, we present in situ cell-surface proteome extraction by extracellular labeling (iPEEL), a proximity labeling method in mice that enables spatiotemporally precise labeling of cell-surface proteomes in a cell-type-specific environment in native tissues for discovery proteomics. Applying iPEEL to developing and mature cerebellar Purkinje cells revealed differential enrichment in CSPs with post-translational protein processing and synaptic functions in the developing and mature cell-surface proteomes, respectively. A proteome-instructed in vivo loss-of-function screen identified a critical, multifaceted role for Armh4 in Purkinje cell dendrite morphogenesis. Armh4 overexpression also disrupts dendrite morphogenesis; this effect requires its conserved cytoplasmic domain and is augmented by disrupting its endocytosis. Our results highlight the utility of CSP profiling in native mammalian tissues for identifying regulators of cell-surface signaling.

## eTOC

Shuster and Li et al. introduce *iPEEL*, a method for cell-type-specific *in situ* cell-surface proteome profiling in mice. Using *iPEEL*, they profile the cell-surface proteomes of developing and mature cerebellar Purkinje cells and identify a multifaceted dendritic morphogenesis function for Armh4, a transmembrane protein preferentially expressed in developing Purkinje cells.

## Graphical Abstract

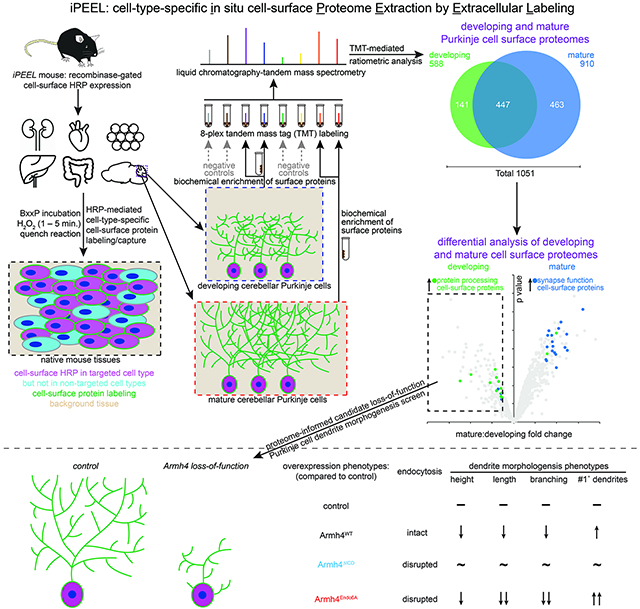

## INTRODUCTION

Complex tissues such as the mammalian nervous system require highly orchestrated interactions between their constituent cell types. Cell-surface proteins (CSPs), including secreted and transmembrane proteins, mediate these interactions throughout the body, from developing embryos to aging organ systems. Accordingly, biochemical identification of CSPs has led to many landmark discoveries, from the identification of peptide hormones to the discovery of regulators of neural development and immune system function (Banting et al., 1922; Brazeau et al., 1973; Cohen et al., 1954; Dinarello et al., 1977; Drescher et al., 1995; Serafini et al., 1994). General methods for profiling cell-surface proteomes would greatly facilitate studies of cell-cell interactions in diverse tissues and physiological states.

CSP profiling has been achieved in dissociated mammalian cells (Bausch-Fluck et al., 2015; Han et al., 2018; Loh et al., 2016; van Oostrum et al., 2020; Pischedda et al., 2014; Sharma et al., 2015; Wollscheid et al., 2009), but such preparations lack the full complement of native cell-cell interactions required for tissue development and function in vivo. Recent approaches have utilized newly engineered proximity labeling enzymes (Branon et al., 2018) to profile proteins at the interface between two cell types (Takano et al., 2020) or proteins in the secretory pathway (Droujinine et al., 2021; Kim et al., 2021; Liu et al., 2021; Wei et al., 2021; Yang et al., 2022), but there have been no general approaches for profiling mammalian cell-surface proteomes in native tissues in a cell-type-specific manner. While recent advances in single-cell RNA sequencing technologies have provided tremendous insight into RNA expression in specific cell types obtained by dissociation of live tissue, transcriptomes and proteomes often correlate modestly at best (Carlyle et al., 2017; Ghazalpour et al., 2011; Gygi et al., 1999; Li et al., 2020; Wang et al., 2019), such that protein levels are difficult to predict from transcriptomes.

Here we present in situ cell-surface proteome extraction by extracellular labeling (iPEEL), which targets a proximity labeling enzyme to the cell surface of specified cell types in transgenic mice for profiling of cell-surface proteomes with spatiotemporal precision. iPEEL is an extension of a similar method we developed in *Drosophila* (Li et al., 2020), which enabled the discovery of new wiring molecules in the fly olfactory circuit (Li et al., 2020) and the demonstration of the combinatorial actions of CSPs in executing the wiring commands of a transcription factor (Xie et al., 2022). We show here that iPEEL allows efficient cell-surface labeling across diverse mammalian tissues. Applying iPEEL to profile CSPs of cerebellar Purkinje cells, we found different classes of CSPs selectively enriched in developing and mature cerebellar Purkinje cells, despite substantial overlap in the most highly enriched CSPs at both timepoints. Our proteome data allowed us to identify candidate CSPs with potential roles in Purkinje cell dendrite morphogenesis. In-depth analysis of Armadillo-like helical domain-contain protein 4 (Armh4), a protein with no known function in the nervous system, revealed its critical, multifaceted role in Purkinje cell dendrite morphogenesis.

## RESULTS

### In situ cell-surface proteome labeling

**Figure 1.**
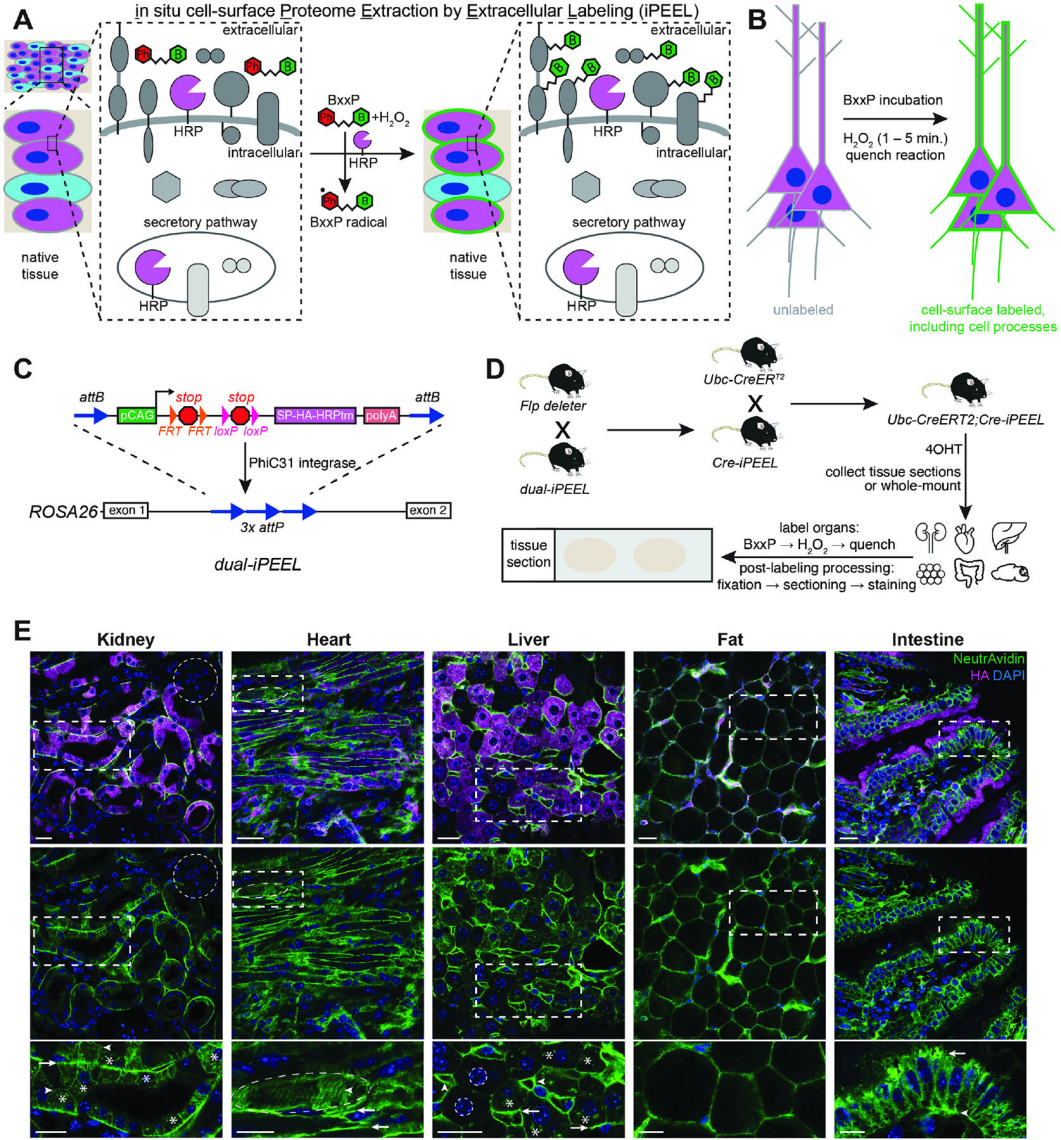
Schematic of iPEEL and its characterization in various tissues (A) Schematic of iPEEL. In the presence of H_2_O_2_ and BxxP (a membrane impermeable biotin substrate), a plasma membrane-targeted, extracellular-facing horseradish peroxidase (HRP) catalyzes transfer of biotin onto nearby extracellular residues of CSPs. Magenta, cells expressing HRP; cyan, cells not expressing HRP; green, CSPs labeled with biotin. (B) Schematic of labeling surfaces of cells with complex morphologies in tissue. (C) Integrase-mediated transgenesis of targeting construct for cell-type-specific expression of extracellularly-facing, HA-tagged, cell-surface HRP. SP, signal peptide; tm, transmembrane domain. (D) Mating scheme to express HRP in diverse tissues for proof-of-principle experiments. (E) NeutrAvidin (green) and HA (magenta) stains in kidney, heart, liver, back skin fat, and intestine, respectively, show that iPEEL-mediated biotinylation is enriched at the cell surface (green, revealed by NeutrAvidin stain) despite intracellular HRP (magenta, HA stain), presumably in the secretory pathway. Bottom row, magnified images of boxed regions in middle row. Arrows indicate tubular basement membrane, cardiomyocyte plasma membrane, hepatocyte basolateral membrane, and enterocyte apical membrane; arrowheads indicate renal tubular epithelial cell plasma membrane, cardiomyocyte t-tubules, canalicular membrane, and enterocyte basal membrane (in kidney, heart, liver, and intestine images, respectively). The oval indicates a cardiomyocyte with many t-tubules labeled. Green puncta near asterisks indicate likely labeled endosomes in renal epithelial cells and hepatocytes. Circles indicate cells lacking HRP expression, due to the mosaic nature of CreER-induced recombination, as judged by a lack of intracellular HA staining and a corresponding lack of Neutravidin signal at the cell surface. The intestinal mucosal layer staining likely results from binding of endogenous Fc*γ*bp to the anti-HA primary antibody (Kobayashi et al., 2002). Scale bars, 20 μm (top and middle rows); 10 μm (bottom row).

iPEEL utilizes a synthetic transmembrane protein with an extracellular portion containing the horseradish peroxidase (HRP) enzyme. HRP catalyzes tagging of CSPs with biotin using BxxP, a membrane-impermeant substrate (Loh et al., 2016), allowing rapid biotinylation of CSPs in live native tissues (Figures 1A and 1B). To enable cell-type specificity, we generated a transgenic mouse expressing membrane-bound, extracellularly-facing, HA-tagged HRP under the control of both Cre and Flp recombinases (Figure 1C). We used integrase-mediated transgenesis (Tasic et al., 2011) to integrate this construct into the globally accessible *ROSA26* genomic locus under the control of the ubiquitously active *CAG* promoter (Muzumdar et al., 2007; Zong et al., 2005).

To test the applicability of iPEEL in native tissues, we bred the resulting Cre- and Flp-dependent (*dual-iPEEL*) mice to *Flp deleter* mice (Farley et al., 2000) to produce Cre-dependent HRP (*Cre-iPEEL*) mice, which we then crossed to *Ubc-CreER^T2^* mice (Ruzankina et al., 2007) with tamoxifen-inducible Cre expression in widespread cell types and tissues in a mosaic fashion. We collected kidney, heart, liver, fat, and intestine tissues, whole-mount or sectioned, and performed proximity labeling reactions (Figure 1D; STAR★Methods). In all organs examined, NeutrAvidin staining signal, reflecting biotinylation, was enriched at the surfaces of cells expressing our HRP transgene, as indicated by concurrent staining of the HA tag (Figure 1E). Cells without HA signal did not display surface staining, while cells with HA signal displayed biotinylation on their surfaces but not in their intracellular compartments, except for occasional endosome-like structures in select cell types. These experiments established the broad applicability of iPEEL across diverse organs and tissues.

### Capturing cell-surface proteomes of cerebellar Purkinje cells

**Figure 2.**
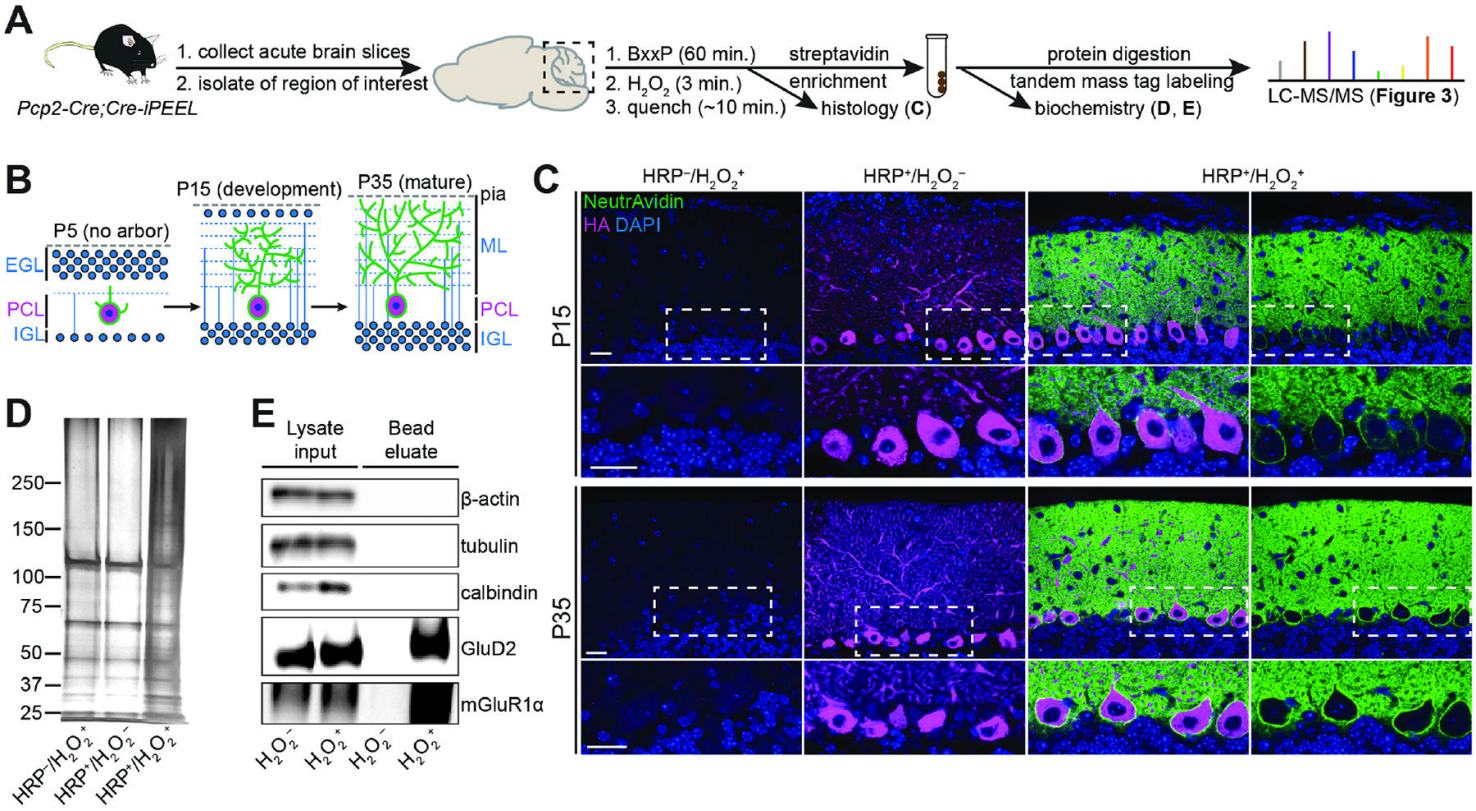
Cell-surface biotinylation of developing and mature cerebellar Purkinje cells (A) Pipeline for profiling Purkinje cell-surface proteomes, including histological and biochemical evaluation en route to proteome capture and analysis by liquid chromatography-tandem mass spectrometry (LC-MS/MS). (B) Purkinje cell postnatal development. During the first postnatal week (e.g., postnatal day 5, P5), Purkinje cells do not have elaborated dendritic arbors. At P15, the Purkinje cell dendritic arbor is still growing. At P35, the Purkinje cell dendritic arbor is fully mature. EGL, external granule cell layer; PCL, Purkinje cell layer; IGL, internal granule cell layer; ML, molecular layer. (C) Representative confocal images of P15 (top) and P35 (bottom) negative control (HRP^−^/H_2_O_2_^+^, HRP^+^/H_2_O_2_^−^) and experimental (HRP^+^/H_2_O_2_^+^) conditions. Staining for NeutrAvidin (green) and HA (magenta) shows biotinylation (green) at the Purkinje cell surface only in experimental conditions. While the HA-tagged HRP enzyme, visualized by anti-HA immunostaining, is also abundant intracellularly (likely due to its localization in the ER, Golgi, and secretory pathways), biotinylation, visualized by NeutrAvidin staining, is highly concentrated on the cell surface due to use of the membrane-impermeable biotin substrate BxxP. The left three columns show triple staining channels; the rightmost column shows NeutrAvidin and DAPI staining only. Scale bars, 30 μm. (D) Silver stain of streptavidin bead-enriched protein fractions of control (HRP^−^/H_2_O_2_^+^, HRP^+^/H_2_O_2_^−^) and experimental (HRP^+^/H_2_O_2_^+^) samples showing marked enrichment of proteins in the experimental, compared to control, condition. Left, molecular weight markers in kilodaltons. (E) Western blots showing the presence of CSPs GluD2 and mGluR1α, but absence of abundant intracellular proteins ß-actin, tubulin, and calbindin, after streptavidin bead enrichment under control (H_2_O_2_^−^) and experimental (H_2_O_2_^+^) conditions. See also Figure S1.

To test the feasibility of using iPEEL to extract CSPs for proteomic analysis via mass spectrometry (MS), we applied it to developing and mature cerebellar Purkinje cells (Figure 2A). Purkinje cells extend expansive, complex, yet morphologically stereotyped dendritic arbors, and are an excellent model for studying molecular control of dendrite morphogenesis. We chose postnatal days 15 (P15) and 35 (P35) for proteomic analysis: P15 Purkinje cell dendritic arbors are still growing, while P35 Purkinje cells are stably integrated into mature cerebellar circuitry (Figure 2B). To generate mice in which HRP is expressed specifically in Purkinje cells, we crossed *Pcp2-Cre* mice (Zhang et al., 2004) to our *Cre-iPEEL* mice. We carried out the cell-surface biotinylation reaction in acute cerebellar slices for 3 minutes, followed by histological and biochemical analyses (Figure 2A). Omitting Cre (and thus HRP expression) or H_2_O_2_ (which initiates HRP-mediated biotinylation) both resulted in undetectable levels of NeutrAvidin staining, while including these components resulted in robust labeling of Purkinje cell somatodendritic surfaces in both developing (P15) and mature (P35) cerebellar tissue (Figures 2C and S1). Biochemical analysis indicated that experimental samples were substantially enriched in biotin-labeled proteins (Figure 2D), and that iPEEL enriched for cell-surface receptors GluD2 and mGluR1 while excluding abundant intracellular proteins β-actin, tubulin, and calbindin (Figure 2E).

**Figure 3.**
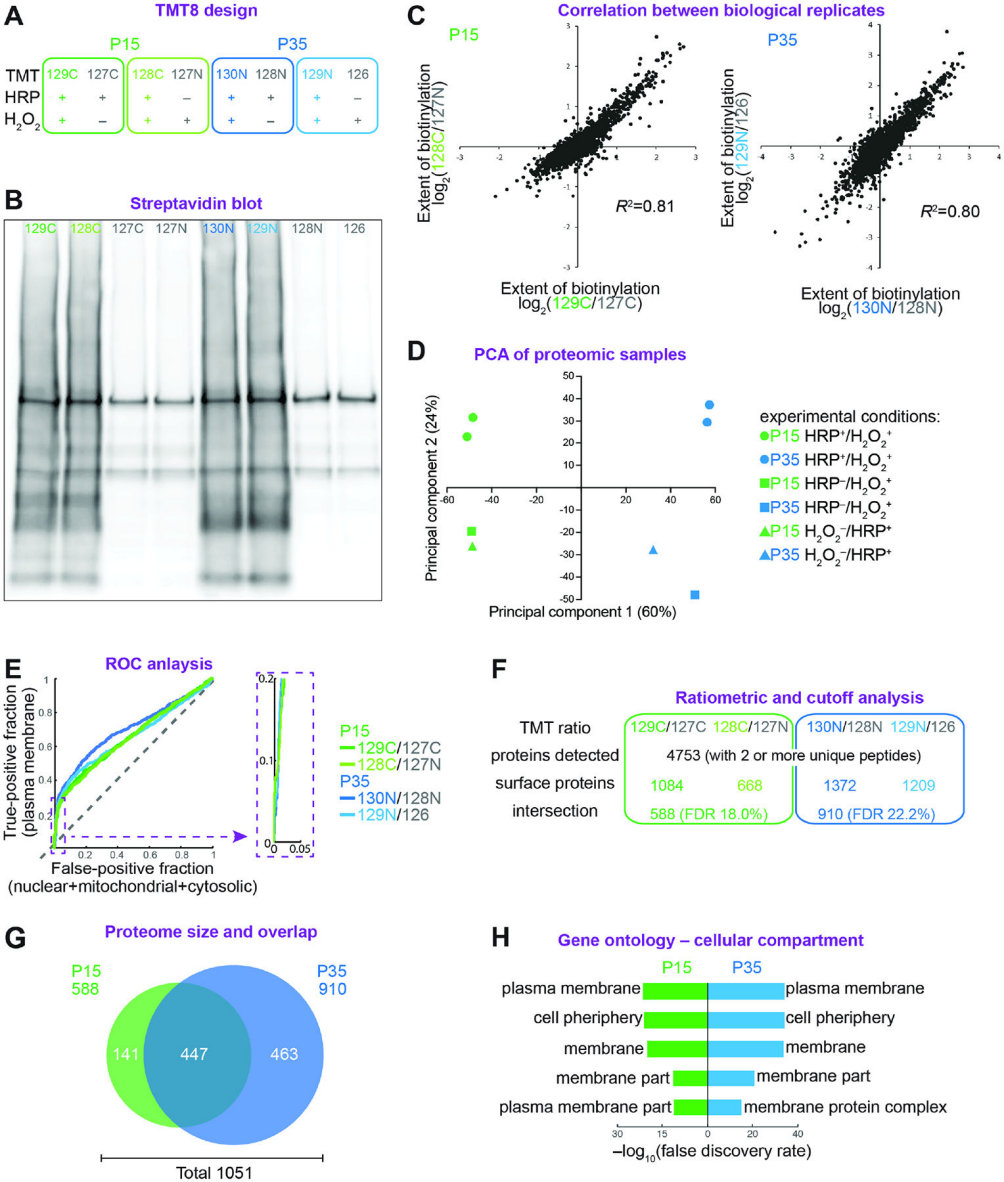
Multiplexed cell-surface proteomic profiling of Purkinje cells using iPEEL (A) 8-plex tandem mass tag (TMT) ratiometric proteomic study design, with pairing of each experimental sample with a negative control. (B) Streptavidin blot of samples for proteomic analysis. Lanes with experimental samples have much stronger signal than lanes with control samples. (C) Biological replicates at both stages exhibit high correlations. (D) Principal component analysis (PCA) of proteomes reveals separated clustering of experimental samples and negative controls at both stages. The first and second principal components represent variation caused by sample stage (PC1; 60% variation) and experimental vs. control conditions (PC2; 24% variation). (E) Receiver operating characteristic (ROC) curve showing proportion of true-positive (plasma membrane localized) and false-positive (nuclear, mitochondrial, or cytosolic) proteins rank ordered (from 0, 0) by enrichment in each ratiometric pair. Annotations were curated by the UniProt database. (F) Ratiometric and cutoff analysis summary. (G) Sizes and overlap of developing and mature Purkinje cell-surface proteomes. (H) Gene ontology analysis of post-cutoff Purkinje cell-surface proteomes reveals enrichment in cellular compartment terms associated with the plasma membrane and cell periphery. See also Figure S2.

Next, we prepared Purkinje cell CSP samples for quantitative liquid chromatography–tandem mass spectrometry (LC-MS/MS). To quantify our MS results and filter out contaminants, we employed a ratiometric tandem mass tag (TMT) strategy (Hung et al., 2014; Thompson et al., 2003), pairing control samples lacking HRP or H_2_O_2_ with one of two experimental biological replicate samples at each stage (Figure 3A). Experimental samples showed much more streptavidin-binding signal (i.e., protein biotinylation) than controls (Figure 3B). Developing and mature samples were processed for proteomic analysis by LC-MS/MS (STAR★Methods). Biological replicates at both stages correlated highly (Figure 3C), and a principal component analysis of all eight samples revealed variation by developmental stage and experimental condition as the first two principal components (Figure 3D). In a receiver operating characteristic analysis, the top 20% most highly enriched proteins yielded almost vertical curves, confirming highly specific enrichment in all pairings (Figure 3E, magnification). To further deplete potential contaminants and maximize the signal-to-noise ratio, we cut off each biological replicate at the maximal value of the true positive rate minus false positive rate and included only proteins present in both replicates at each stage (Figures 3F and S2A). This proteomic analysis yielded 588 and 910 proteins in the developing and mature Purkinje cell-surface proteomes, respectively (Figures 3F and 3G; Table S1). Out of a total of 1051 proteins, 447 proteins were shared by both stages, whereas 604 proteins were stage-specific (Figure 3G). Gene ontology analysis of the cellular compartment of the cell-surface proteomes revealed enrichment of terms associated with the plasma membrane and cell periphery (Figure 3H), indicating high spatial specificity consistent with our histological and biochemical analyses (Figures 2C and 2E). Applying different cutoff methods and more stringent criteria resulted in similar protein localization and function annotations based on gene ontology analysis (Figures S2B-S2D; complete protein lists are provided in Table S1). Thus, these multimodal analyses validated the quality, consistency, and specificity of our developing and mature Purkinje cell-surface proteomes.

Comparison of our cell-surface proteome data to recently published cerebellar cell-type-specific transcriptomes (Buchholz et al., 2020; Kozareva et al., 2021) revealed that while the majority of the top 100 most highly enriched CSPs in our P35 proteomes were expressed in Purkinje cells, their cell-surface protein abundance did not correspond well to their RNA levels (Figure S3A). Aside from the modest correlations between transcriptomes and proteomes often reported (Carlyle et al., 2017; Ghazalpour et al., 2011; Gygi et al., 1999; Li et al., 2020; Wang et al., 2019), cell-type-specific transcriptomes are typically extracted from somata or nuclei, whereas our cell-surface proteomes were extracted from the surfaces of Purkinje cells, whose dendritic surface area is around two orders of magnitude larger than their somatic surface area and whose dendrites contain mRNAs and ribosomes (Bian et al., 1996; Kratz et al., 2014). Indeed, some CSPs identified by iPEEL had very low nuclear RNA levels (Kozareva et al., 2021) but higher mRNA levels detected by translating ribosomal affinity purification (Buchholz et al., 2020), which can detect dendritic mRNAs, suggesting the possibility of dendritic translation of these CSPs (Figure S3A). Our proximity labeling-based approach is also expected to label CSPs produced in both the cell type expressing HRP and cell types that physically contact the HRP-expressing cell type (Figure S3B), given an estimated labeling radius of 10 nm (Qin et al., 2021). Moreover, secreted proteins could diffuse over longer distances, enriching at surfaces of non-expressing cells through ligand-receptor interactions. Indeed, some of the top 100 most highly enriched P35 Purkinje cell CSPs were more highly expressed in nearby cell types than in Purkinje cells (Figures S3A-S3E). We also estimated the coverage of iPEEL, which detected a majority of CSPs Purkinje cells are predicted to express based on transcriptome data (Figure S3F). In summary, cell-surface proteome analysis complements RNA-sequencing studies by providing a more direct readout of proteins in the extracellular milieu of specific cell types in densely interconnected neural tissues.

### Comparing developing and mature Purkinje cell-surface proteomes

**Figure 4.**
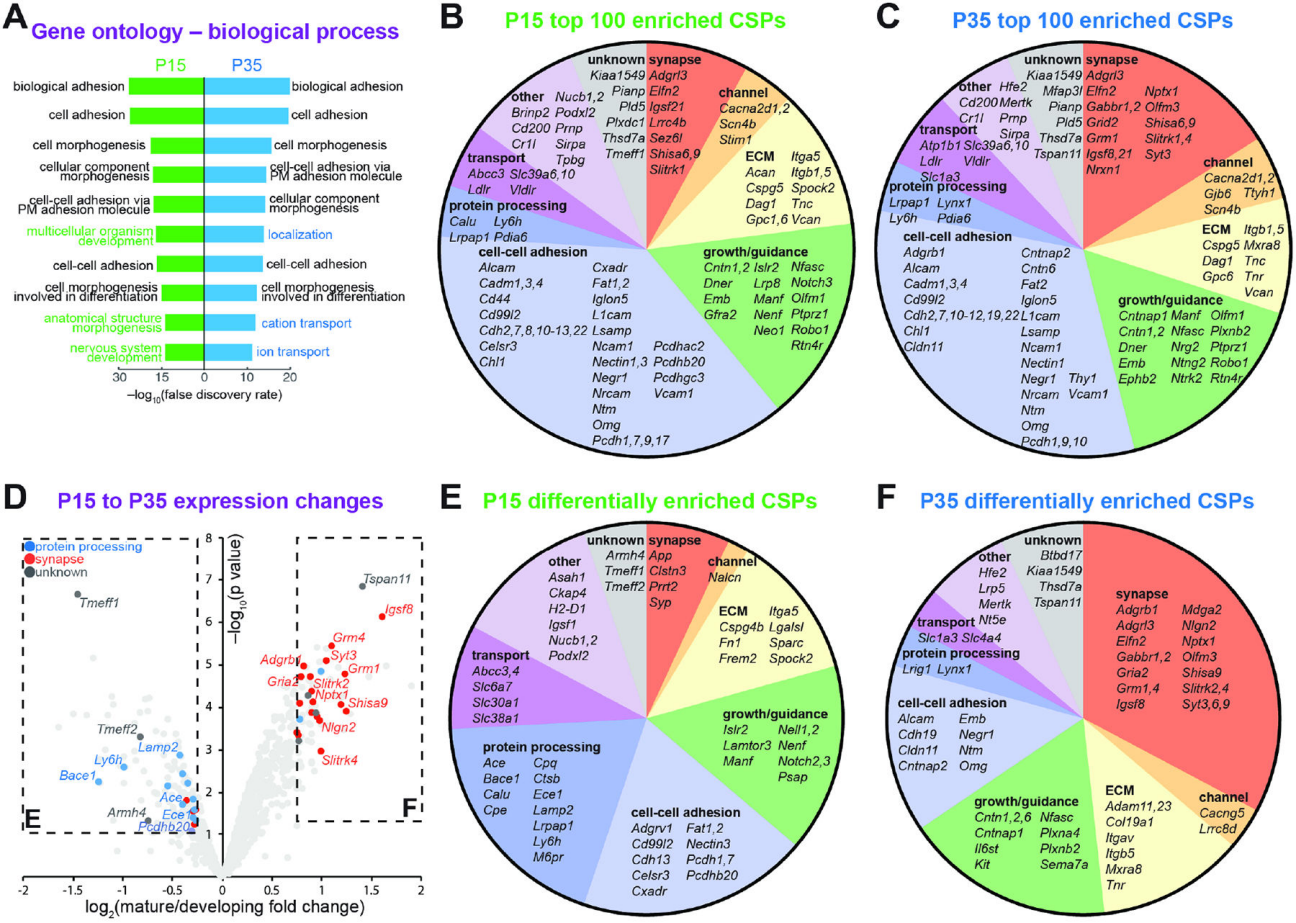
Developing and mature cerebellar Purkinje cell-surface proteomes (A) Gene ontology analysis of Purkinje cell-surface proteomes reveals enrichment in biological process terms associated with cell adhesion and morphogenesis (black). The P15 proteome also enriched in developmental terms (green) while the P35 proteome enriched in terms relating to ion transport (blue). PM, plasma membrane. (B and C) Top 100 enriched CSPs of P15 (B) and P35 (C) Purkinje cells categorized by primary function. Note that many CSPs can belong to multiple functional categories—e.g., cell-cell adhesion, neuronal process growth and guidance (growth/guidance), synapse function—but for simplicity, we placed each CSP into only one category based on its best described functions in UniProt (https://www.uniprot.org). ECM, extracellular matrix. (D) Volcano plot showing differentially enriched CSPs. Each dot represents one CSP. CSPs associated with synapse function (red) are enriched in the P35 proteome, whereas those associated with posttranslational protein processing (blue) are enriched in the P15 proteome. A subset of proteins color coded following the categorization scheme in (E) and (F) are highlighted. (E and F) Lists of P15 (E) and P35 (F) differentially enriched CSPs of from the rectangles in (D), categorized by primary function (see Figure S4 for more details). ECM, extracellular matrix. See also Figures S2-S4.

Gene ontology analysis of biological processes revealed uniform enrichment in terms associated with cell adhesion and morphogenesis (Figure 4A, black), with the developing sample associated more with developmental terms (Figure 4A, green) and the mature sample associated more with physiological and homeostatic terms such as ion transport (Figure 4A, blue). Further analysis of the cell-surface proteomes at each stage revealed roughly similar proportions of functional modules in the 100 most enriched proteins (Figure 4B and 4C), including proteins with primary functions in synaptic transmission, neuronal process growth and guidance, extracellular matrix, and cell-cell adhesion. Indeed, the top 100 most enriched CSPs of the two proteomes share 66 proteins (Figures 4B, 4C, and S4). Notably, a substantially higher proportion of CSPs with functions associated with the extracellular matrix were detected here compared to studies using cultured neurons (Loh et al., 2016), emphasizing the importance of profiling cell-surface proteomes from native tissues. This analysis also revealed the presence of many of the same synaptic and channel proteins at both stages, highlighting the active engagement of electrical and synaptic signaling in developing postnatal Purkinje cells well before the establishment of mature circuitry.

The detection levels of many CSPs changed profoundly between P15 and P35 (Figure 4D). Analysis of the most differentially enriched CSPs at each stage revealed marked enrichment in the mature Purkinje cell-surface proteome of CSPs with synaptic functions (Figures 4D-4F), including ionotropic and metabotropic neurotransmitter receptors (e.g., *Gabbr1, Gabbr2, Gria2, Grm1, Grm4*), neurotransmitter release machinery (e.g., *Syt3, Syt6, Syt9*), synaptic adhesion molecules (e.g., *Adgrl3, Nlgn2*), and a neurotransmitter transporter (*Slc1a3*). This analysis suggests the selective upregulation and expansion of a basic repertoire of synaptic proteins over the course of neuronal maturation. Conversely, the developing Purkinje cell-surface proteome selectively enriched CSPs with functions in posttranslational protein processing such as proteolytic enzymes (e.g., *Ace, Bace1, Cpe, Cpq, Ece1*) and regulators of protein trafficking (e.g., *Lrpap1, Ly6h*), consistent with a previous transcriptomic study of developing *Drosophila* central nervous system neurons (Xie et al., 2021), suggesting that developing neuronal cell-surface proteomes are more dynamic. Furthermore, despite the abundance of cell adhesion proteins in both proteomes, including many members of the cadherin and immunoglobulin superfamilies of CSPs, clustered protocadherins stood out as enriched in the developing proteome (Figures 4B, 4D, and 4E), consistent with previous reports that clustered protocadherins regulate self-avoidance in growing Purkinje cell dendrites (Ing-Esteves et al., 2018; Lefebvre et al., 2012). These results suggest that iPEEL can detect salient CSPs during development.

### Purkinje cell dendrite morphogenesis requires Armh4

**Figure 5.**
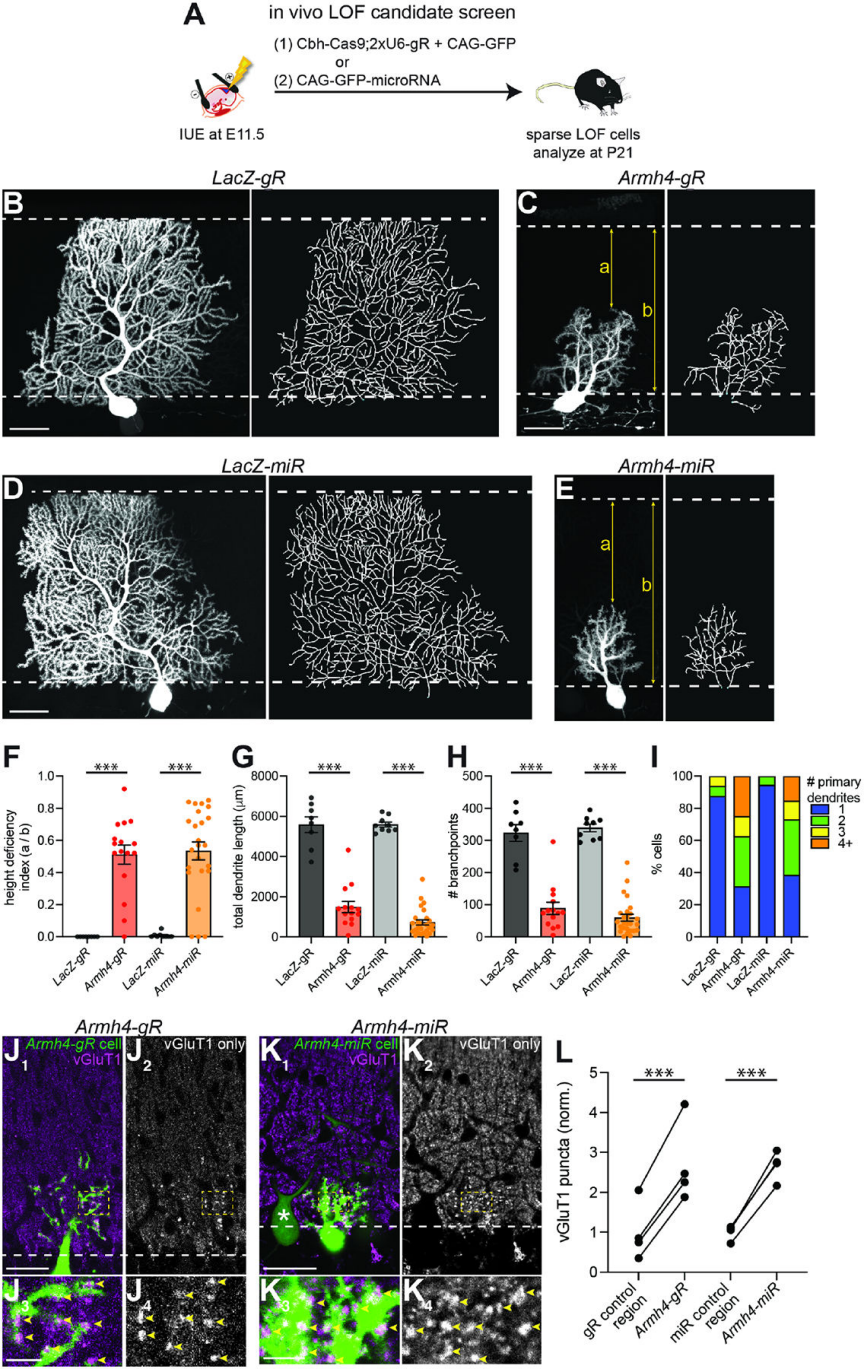
An in vivo loss-of-function screen of proteomic candidates reveals a critical, multifaceted role for Armh4 in Purkinje cell dendrite development (A) Schematic of in vivo loss-of-function (LOF) screen for regulators of Purkinje cell dendrite development via in utero electroporation (IUE). Plasmids encoding Cas9, guide RNAs (gR), and GFP (1) or plasmids encoding both GFP and a microRNA (miR) (2) were electroporated into newborn Purkinje cells at embryonic day 11.5 (E11.5). Phenotypes were analyzed at postnatal day 21 (P21). (B, D) Control Purkinje cells (left, confocal image; right, trace) expressing Cas9/gRs (B) or a miR (D) targeting *lacZ*, a bacterial gene, extend one primary dendrite, elaborate widely throughout the entire depth of the molecular layer (demarcated by dashed white lines), and reach the pial surface. (C, E) Purkinje cells (left, confocal image; right, trace) expressing Cas9/gRs (C) or a miR (E) targeting *Armh4* exhibit supernumerary primary dendrites and stunted dendrite growth and fail to reach the pial surface. (F–I) Quantification of height deficiency index (F), total dendrite length (G), number of branchpoints (H), and number of primary dendrites (I) of control and *Armh4 LOF* Purkinje cells. Data are mean ± SEM from 2 mice each; for (F), *n* = 8, 16, 9, and 24 cells for the four genotypes from left to right, respectively; for (G) and (H), *n* = 8, 14, 9, and 26 cells; for (I), *n* = 16, 16, 18, and 26 cells. *p* values were calculated using one-way ANOVA followed by Sidak’s multiple comparisons test. Adjusted *p* values: ***p < 0.001. (J, K) Single-plane confocal images of P21 *Armh4-gR* (J) and *Armh4-miR* (K) Purkinje cells (green, left) and vGluT1 immunostaining (magenta, left; white, right). Numerous large, bright vGluT1 puncta (arrowheads) abut *Armh4 LOF* Purkinje cell dendritic processes but are less abundant in nearby regions occupied by unlabeled wild-type Purkinje cell dendrites. Asterisk marks low *Armh4-miR*-expressing cell. (L) Quantification of the number of large vGluT1 puncta per molecular layer area (normalized to nearby control regions). Data are from n = 4 gR control regions paired with 4 *Armh4-gR* dendritic regions and 4 miR control regions paired with 4 *Armh4-miR* dendritic regions, *p* values were calculated using paired t-tests followed by a Bonferroni correction. Adjusted *p* values: ***p < 0.005. Scale bars, 30 μm for (B–E, J_1,2_, K_1,2_), 10 μm for insets in (J_3,4_, K_3,4_). See also Figure S5.

To discover new cell-surface regulators of dendrite morphogenesis, we developed an in vivo loss-of-function (LOF) screen of candidates from our cell-surface proteomes. Candidates were selected based on their enrichment in our developing Purkinje cell-surface proteome, mRNA expression in Purkinje cells (Lein et al., 2007; Saunders et al., 2018; Zeisel et al., 2018) (Figure S3), and no known role in dendrite development. To simultaneously disrupt gene function in newborn Purkinje cells and label the same cells for morphological analysis, we performed in utero electroporation at embryonic day 11.5, transfecting plasmids encoding Cas9, guide RNAs (gR), and GFP, or a microRNA (miR) and GFP (Figure 5A) (Nishiyama et al., 2012; Takeo et al., 2021). Analysis of 13 candidates using gR- and/or miR-based targeting suggested several CSPs with possible roles in Purkinje cell dendrite morphogenesis (Table S2).

We focused our analysis on Armadillo-like helical domain-containing protein 4 (Armh4), a protein enriched in the developing Purkinje cell-surface proteome (Figures 4D and 4E), as *Armh4* LOF using both gR- and miR-based methods yielded the strongest phenotypes we observed. Antibody staining confirmed loss of endogenous Armh4 protein in *Armh4 LOF* Purkinje cells (Figures S5A and S5B). Armh4 is a type-I transmembrane protein implicated in regulating cell proliferation in the context of stem cells and cancer (Lee et al., 2014, 2016) but has no described roles in the nervous system. *Armh4* mRNA is highly enriched in Purkinje cells (Figures S3E and S5C). Compared to controls (Figures 5B, 5D, S5F, and S5H), P21 *Armh4 LOF* Purkinje cells displayed drastically stunted dendrite growth (Figures 5C, 5E, S5G, and S5I), including failure to reach the pial surface by P21 (Figure 5F), substantially decreased total dendrite length and branching (Figure 5G and 5H), and supernumerary primary dendrites (Figure 5I). Similar dendritic phenotypes were also observed in P42 *Armh4 LOF* Purkinje cells (Figures S5J and S5K), suggesting that they did not result from developmental delay or cell toxicity.

To further probe for potential roles of Armh4 in neuronal development, we stained cerebellar sections with *Armh4 LOF* cells with an antibody against vesicular glutamate transporter 1 (vGluT1), a marker of parallel fiber→Purkinje cell synapses. P21 *Armh4-gR* and -*miR LOF* Purkinje cells had more large, bright vGluT1 puncta abutting their dendrites than control regions in the cerebellar molecular layer (Figure 5J-L). The sparseness of these LOF manipulations suggests that disrupting Armh4 in Purkinje cells leads to impaired parallel fiber→Purkinje cell synapse formation via retrograde transsynaptic signaling from Purkinje cell dendrites to presynaptic parallel fiber axons.

### Armh4 cell-surface levels regulate dendrite morphogenesis

**Figure 6.**
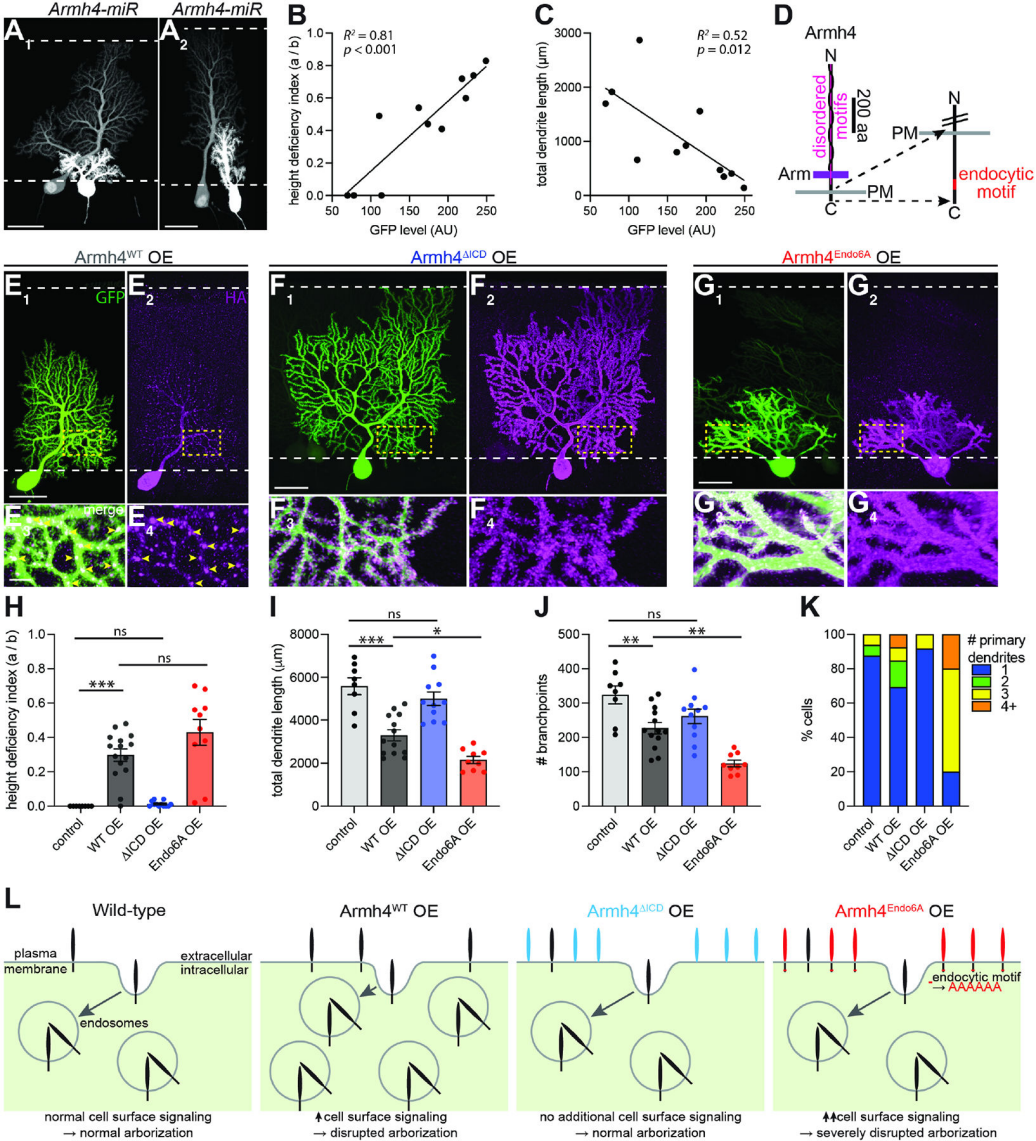
Purkinje cell dendrite morphogenesis requires proper levels of Armh4 cell-surface signaling (A) Confocal images with multiple *Armh4-miR* Purkinje cells. Cells with more GFP (and thus more *Armh4-miR*) have stronger morphological defects. (B and C) Correlations between GFP levels (a proxy for miR levels) and dendrite morphogenesis measures from images with fields-of-view in which multiple *Armh4-miR* Purkinje cells were present. AU, arbitrary unit. See also Figures S5D and S5E. (D) Left, schematic of Armh4. N and C, N- and C-termini; Arm, Armadillo-like domain; aa, amino acids; PM, plasma membrane. Right, schematic of Armh4 peri-transmembrane and intracellular region. Red, endocytic motif. (E–G) Representative confocal images of Purkinje cells sparsely overexpressing GFP and HA-tagged Armh4^WT^ (E), Armh4^ΔICD^ (F), or Armh4^Endo6A^ (G). Top, images of whole dendritic arbors; bottom, magnified images (from dashed yellow boxes) showing subcellular localization of overexpressed Armh4 variants; dashed white lines, top and bottom of cerebellar molecular layer. Yellow arrowheads (E_3_ and E_4_), intracellular Armh4^WT^-HA puncta. (H–K) Quantification of height deficiency index (H), total dendrite length (I), number of branchpoints (J), and number of primary dendrites (K) of control (*lacZ-gR* from Figure 5) and Armh4^WT^, Armh4^ΔICD^, and Armh4^Endo6A^ overexpressing Purkinje cells. Data are mean ± SEM; for (H), n = 8, 15, 12, and 10 cells from 2, 2, 2, and 4 mice for the four conditions from left to right, respectively. For (I) and (J), n = 8, 13, 11, and 9 cells. For (K), n = 16, 13, 12, and 10 cells. *p* values were calculated using one-way ANOVA followed by Sidak’s multiple comparisons test. Adjusted *p* values: *p < 0.05, **p < 0.01, ***p < 0.001. Scale bars, 5 μm for insets of (E–G); 30 μm for all others. (L) Schematic interpretation of Armh4 variant overexpression results. Under normal conditions (wild-type), the cell-surface level of WT Armh4 (black) is downregulated by endocytosis. Armh4^WT^ overexpression (OE) leads to increased Armh4 cell-surface levels and increased signaling, resulting in disrupted dendrite arborization. Armh4^ΔICD^ OE (blue) does not disrupt dendrite arborization despite a higher level of cell-surface expression, as the cytoplasmic domain is required for signaling. Armh4^Endo6A^ OE (red) leads to severely disrupted dendrite arborization, likely because Armh4^Endo6A^ does not undergo proper endocytosis, leading to even more cell-surface signaling than Armh4^WT^ OE. See also Figures S5-S8.

Purkinje cell dendrite morphogenesis appears to be highly sensitive to Armh4 levels based on the following lines of evidence. First, analysis of miR-based LOF experiments revealed correlations between phenotypic severity and the level of co-expressed GFP (Figures 6A-6C, S5D, and S5E). Second, overexpression of wild-type (WT) Armh4 using in utero electroporation (Figures 6E and S6B) also caused dendrite morphology phenotypes, including failure to reach the pial surface (Figure 6H), decreased total dendrite length (Figure 6I) and branching (Figure 6J), and supernumerary primary dendrites (Figure 6K). These data imply that a precise level of Armh4 signaling is necessary for appropriate elaboration of Purkinje cell dendrites and highlight the importance of precise levels of CSPs in proper neural wiring (Li et al., 2018; Takeo et al., 2021).

To gain insight into the mechanisms underlying Armh4 signaling in dendrite morphogenesis, we performed structure-function analysis using an overexpression assay. We focused on Armh4’s intracellular domain (Figure 6D), which is highly conserved across vertebrates (Figure S6A). Overexpression of Armh4 lacking its C-terminal intracellular domain (ΔICD) did not cause any morphological phenotypes (Figures 6F, 6H-6K, and S6C), indicating that signaling through its intracellular domain is essential for Armh4 regulation of dendrite morphogenesis.

Notably, Armh4^WT^ and Armh4^ΔICD^ were enriched in different subcellular compartments in Purkinje cell dendrites. Armh4^WT^ localized strongly to intracellular puncta in dendrites (Figures 6E_3_, 6E_4_, and S7A), as did endogenous Armh4 protein (Figures S5A and S5B), suggestive of endolysosomal localization. This is consistent with the presence of a conserved endocytic motif in Armh4’s intracellular domain (Figures 6D and S6A) (Owen et al., 2004) and partial co-localization of Armh4 intracellular puncta with endolysosomal markers (Figure S8). By contrast, Armh4^ΔICD^ was present throughout the dendritic surface and enriched in dendritic spines, but not in large intracellular puncta (Figures 6F_3_, 6F_4_, and S7B). This postsynaptic localization is consistent with a role in synapse regulation suggested by the vGluT1 analysis (Figure 5J-5L).

Due to this divergence in subcellular localization, we investigated the role of Armh4 localization on Armh4 function. Overexpression of an Armh4 mutant with its six amino acid endocytic motif changed to alanines (Armh4^Endo6A^; Figure 6G), which was more localized to the dendritic surface than Armh4^WT^ (Figures 6G_3_, 6G_4_, S7C, and S7D), resulted in an even stronger phenotype than overexpression of Armh4^WT^ (Figures 6G-6K and S6D). These data suggest that the intracellular domain is required for signaling (Figures 6E and F) and that inhibiting endocytosis and thus elevating cell-surface levels increases signaling (Figures 6E and G). While multiple interpretations could follow from these observations, a parsimonious interpretation is that Armh4 signals primarily from the Purkinje cell plasma membrane and that its endocytosis constitutes posttranslational tuning for precise levels of cell-surface signaling (Figure 6L).

## DISCUSSION

Here we report a flexible approach for profiling cell-type-specific cell-surface proteomes in mouse tissues with spatiotemporal precision. Using this approach, we describe the cell-surface proteomes of developing and mature cerebellar Purkinje cells, lending insight into how the neuronal surface milieu evolves over development. We also performed a proteome-directed in vivo screen of candidate regulators of dendrite morphogenesis. We identified a critical role for Armadillo-like helical domain-contain protein 4 (Armh4) in Purkinje cells and showed that endocytosis tunes Armh4 cell-surface levels and impacts dendrite morphogenesis. These results exemplify the potential of cell-surface proteomic profiling in native tissues for determining critical changes in cell-surface protein (CSP) repertoires under different experimental conditions and identifying new regulators of cell-surface signaling events.

### iPEEL: a flexible method for cell-type-specific, temporally resolved cell-surface proteome profiling in mammalian tissues

Proteomic profiling constitutes a powerful class of methods for both characterizing proteomes and identifying key regulators of biological processes in complex tissues like the mammalian brain (Hosp and Mann, 2017; Sharma et al., 2015). These tissues require intricate, tightly regulated interactions between constituent cell types mediated by CSPs, including secreted, lipid-anchored, and transmembrane proteins. While chemical labeling methods have allowed enrichment of CSPs (Bausch-Fluck et al., 2015; Jang and Hanash, 2003; Loh et al., 2016; Nunomura et al., 2005; van Oostrum et al., 2020; Wollscheid et al., 2009; Zhang et al., 2003), they do not provide cell-type specificity in heterogeneous tissues. Recent advances featuring cell-type specificity have focused on profiling proteins that pass through the secretory pathway (“secretomes”) by targeting biotin ligase variants to the endoplasmic reticulum (ER) lumen (Droujinine et al., 2021; Kim et al., 2021; Liu et al., 2021; Wei et al., 2021; Yang et al., 2022). Even if labeling enzymes were targeted to the cell surface rather than the ER lumen, pools of enzyme would remain in the ER-Golgi network en route to the cell surface and therefore trigger substantial labeling within the ER/Golgi lumen. Another study used a split biotin ligase (split-TurboID) strategy to profile proteins at cell-cell interfaces (Takano et al., 2020). One caveat with this strategy is that the extracellular ATP concentration in healthy tissue is estimated to be <1 μM (Pellegatti et al., 2008), well below the estimated *K*m value of TurboID for ATP (1 mM is typically used). Moreover, the N-terminal split-TurboID fragment used (amino acids 1–256) has intrinsic biotinylation activity (Cho et al., 2020; Takano et al., 2020), raising the possibility that this strategy could include for proteins localized to the secretory pathway of one of the two cell types. In contrast to these recent approaches, iPEEL selectively labels cell-surface proteomes because it uses cell-surface-targeted HRP in combination with BxxP (Loh et al., 2016), a membrane-impermeant biotin substrate, eliminating labeling of intracellular proteins in the ER or Golgi.

iPEEL has several advantages compared to previous cell-surface proteome profiling methods. First, iPEEL allows proteome profiling in native tissues, as opposed to acutely isolated cells (Sharma et al., 2015) or primary cultures (Bausch-Fluck et al., 2015; Han et al., 2018; Loh et al., 2016; van Oostrum et al., 2020; Pischedda et al., 2014; Sharma et al., 2015; Wollscheid et al., 2009). Second, iPEEL features recombinase-gated (Cre, Flp, or both) transgenic strategies for control of HRP expression by many well characterized cell-type-specific Cre and/or Flp drivers. A transgenic approach allows superior consistency and access compared to viral transduction (Kim et al., 2021; Takano et al., 2020; Wei et al., 2021), which is limited by availability of reliable cell-type-specific promoters (Wang et al., 2021) and difficulty accessing some organs and developmental stages. Third, iPEEL labeling only requires a few minutes and thus provides superior temporal resolution compared to biotin ligase-based approaches for proximity labeling in mammals, which require several days for labeling (Droujinine et al., 2021; Kim et al., 2021; Liu et al., 2021; Takano et al., 2020; Wei et al., 2021; Yang et al., 2022). This speed enables studies addressing the effects of acute physiological stimuli and rapid developmental changes. However, compared to biotin ligase-based methods, which can be performed in vivo, a limitation of iPEEL is the need to perform labeling in acute ex vivo explants to allow BxxP to penetrate tissue. While damage from tissue excision can be reduced by following procedures such as those used to prepare acute brain slices for physiological recording studies, development of methods allowing rapid cell-surface labeling in vivo would expand the power of cell-surface proteomic profiling.

The importance of CSP signaling is highlighted by the fact that most drugs approved for treating human diseases target CSPs, especially transmembrane proteins (Christopoulos, 2002; Yin and Flynn, 2016). Previous ground-breaking biochemical discovery of CSPs was biased towards secreted and lipid-anchored proteins due to their relative ease of purification compared to transmembrane proteins (Banting et al., 1922; Brazeau et al., 1973; Cohen et al., 1954; Dinarello et al., 1977; Drescher et al., 1995; Serafini et al., 1994). iPEEL, however, excels at capturing CSPs with no known biases towards secreted or transmembrane proteins. The top 100 most enriched CSPs in our proteome data include CSPs of all molecular classes (Figure S4 and Table S1), with >70% being transmembrane proteins (Figure S4).

Protein tagging by proximity labeling is subject to a few known biases: peroxidase enzymes (HRP/APEX derivatives) generate radicals that label proteins at certain amino acid residues (e.g., tyrosine, tryptophan, cysteine, histidine), while biotin ligase derivatives label at only lysines. Both classes of proximity labeling enzymes generally preferentially label larger proteins due to their having more of these residues, although labeling also depends on protein conformation and residue accessibility. In our study using Biotin-xx-Phenol (BxxP), H_2_O_2_-mediated HRP catalysis induces formation of Biotin-xx-Phenoxyl radicals, which preferentially bond to electron-rich residues (Loh et al., 2016; Rhee et al., 2013). iPEEL is thus expected to more efficiently label proteins with (1) higher percentages of labelable residues, (2) higher percentages of exposed residues, and (3) high expression levels/protein copy number. Subsequent MS analysis requires proteolysis into peptides, such that longer proteins will on average be more highly represented in the resulting MS datasets. Thus, HRP-mediated proximity labeling of extracellular residues of CSPs is not stoichiometric. Nevertheless, proximity labeling-based proteomic approaches have yielded tremendous insight into cellular and subcellular proteomes (Qin et al., 2021).

### Armh4 critically regulates multiple aspects of Purkinje cell dendrite morphogenesis

Neural circuit assembly comprises many interwoven processes, such as axon guidance and dendrite morphogenesis, each critically requiring CSPs (Jan and Jan, 2010; Kolodkin and Tessier-Lavigne, 2011; Sanes and Zipursky, 2020; Zipursky and Sanes, 2010). Dendrites develop specialized morphologies to receive and integrate distinct patterns of synaptic inputs and play a central role in neural computation (London and Häusser, 2005; Stuart and Spruston, 2015); however, dendrite morphogenesis has been less well studied than other neurodevelopmental processes such as axon guidance, particularly in the mammalian brain. Modern approaches combined with mechanistic study of model dendrites such as those of the cerebellar Purkinje cell may shed light on the cellular and molecular principles governing dendrite morphogenesis.

Purkinje cell dendrite morphogenesis occurs over multiple stereotyped phases (Altman, 1972). During the first postnatal week, rodent Purkinje cells have multiple dendritic processes emanating from the cell bodies, none of which is extensively elaborated (Altman, 1972; McKay and Turner, 2005). These are usually pruned down to a single primary dendrite in an activity-dependent manner during the second postnatal week (Bosman and Konnerth, 2009; Hashimoto and Kano, 2005), by which time lower-order dendrites of each Purkinje cell are innervated by a single climbing fiber (an axon of a neuron from the inferior olive in the medulla). Over the same time period, higher-order branches begin elaborating and forming nascent parallel fibers (axons of cerebellar granule cells) (Altman, 1972). This elaboration of higher-order branches is regulated by interactions between Purkinje cells and parallel fibers, their major presynaptic partners (van der Heijden and Sillitoe, 2021; Joo et al., 2014; Park et al., 2019; Takeo et al., 2021). Elaboration of higher-order dendritic branches, which account for most of the dendrite length in mature Purkinje cells, continues during the second and third postnatal weeks, with these arbors reaching the pial surface and thus achieving their full height around P21 in mice.

Disruption of Armh4 signaling, by either LOF or overexpression, impairs multiple dendritic morphogenesis processes described above, including consolidation of a single primary dendrite, extension of dendrites, and elaboration of higher-order dendritic branches (Figures 5 and 6). Indeed, the Armh4 LOF phenotypes appear more severe than those resulting from disruption of any other single molecule previously reported, revealing Armh4 to be a critical regulator of multiple aspects of Purkinje cell dendrite morphogenesis. The localization of Armh4^ΔICD^ and Armh4^Endo6A^ to dendritic spines (Figures 6F, 6G, and S7B-D) and the overaccumulation of vGluT1 signal abutting Armh4 LOF cells (Figures 5J-L) suggest the possibility that Armh4 could interface with synaptic signaling and thus could connect synaptogenesis to dendrite morphogenesis (Takeo et al., 2021). Our structure-function analysis reveals the importance of its highly conserved cytoplasmic domain for signaling and endocytosis for tuning not only its localization but also its signaling levels. Previous work has suggested that Armh4 regulates mTOR and STAT signaling in the context of cell growth (Lee et al., 2014, 2016). Future investigation into the extracellular partners Armh4 may interact with and whether Arhm4 acts through mTOR, STAT, or other signaling pathways in neurons will deepen our understanding of molecular control of dendrite morphogenesis.

## STAR★METHODS

### RESOURCE AVAILABILITY

#### Lead contact

Further information and requests for resources and reagents should be directed to the Lead Contact, Liqun Luo (lluo@stanford.edu).

#### Materials availability

All unique reagents generated in this study are available from the Lead Contact. *iPEEL* mice are available at the Jackson Laboratory under the stock numbers 037697 (*dual-iPEEL*) and 037699 (*Cre-iPEEL*). The targeting construct for use with integrase-mediated transgenesis will be available at Addgene.

#### Data and code availability

The original and processed proteomic data are provided in Table S1. The original mass spectra and the protein sequence database used for searches have been deposited in the public proteomics repository MassIVE (http://massive.ucsd.edu) and are accessible at ftp://MSV000088506@massive.ucsd.edu.

### EXPERIMENTAL MODEL AND SUBJECT DETAILS

#### Mice

All animal procedures followed animal care guidelines approved by Stanford University’s Administrative Panel on Laboratory Animal Care. All mice used in proximity labeling, biochemical, and proteomic experiments were maintained on a C57BL/6 background. Dendrite morphogenesis studies were conducted using *wild-type* timed pregnant CD1 dams ordered from Charles River Laboratories. *Pcp2-Cre* (Zhang et al., 2004), *Flp deleter* (Farley et al., 2000), and *Ubc-CreER^T2^* (Ruzankina et al., 2007) mice were obtained from The Jackson Laboratory. Male mice were used for all proximity labeling, biochemical, and proteomic experiments. Mice of both sexes were used for dendrite morphogenesis studies.

To generate the iPEEL mouse, standard cloning procedures were used to construct a plasmid with *pCAG-FRT-stop-FRT(FSF)-loxP-stop-loxP(LSL)-SP-HA-HRPtm-WPRE-bGHpolyA* flanked by *attB* sites for integrase-mediated transgenesis (Tasic et al., 2011); this cassette was thus integrated into the first intron of *ROSA26*. The *CAG* promoter, *FSF*, and *LSL* cassettes enable high levels of HRP expression gated by Flp and Cre recombinases. The HRP coding DNA sequence was preceded by an IgK signal peptide (SP) and a hemagglutinin (HA) tag and followed by a PDGFRß transmembrane domain (Loh et al., 2016) and a short intracellular domain with Kir2.1 trafficking and ER transport signals separated by GSG linkers. The whole coding sequence was codon optimized and followed by a woodchuck hepatitis virus posttranscriptional regulatory element (*WPRE*) and a bovine growth hormone polyadenylation signal (*bGHpolyA*). The *pCAG-FSF-LSL* and *WPRE-bGHpolyA* cassettes were PCR-amplified from the Ai65 targeting construct (Madisen et al., 2015) and cloned into a plasmid backbone derived from the pBT378 plasmid (Tasic et al., 2011). Integrase-mediated transgenesis was performed by the Stanford Transgenic, Knockout and Tumor Model Center.

We refer to the mice carrying the full *pCAG-FRT-stop-FRT(FSF)-loxP-stop-loxP(LSL)-SP-HA-HRPtm-WPRE-bGHpolyA* transgene integrated at the *ROSA26* locus as *dual-iPEEL* mice (dually gated by Flp and Cre), and mice in which the stop cassette between the two *FRT* sites was deleted in the germline as *Cre-iPEEL* mice (gated only by Cre).

### METHOD DETAILS

#### Proximity labeling in acute brain slices

At postnatal days 15 or 35, *Pcp2-Cre;Cre-iPEEL* mice were anesthetized by exposure to isoflurane and their brains were quickly dissected out and placed in ice-cold carbogenated (5% CO_3_, 95% O_3_) artificial cerebrospinal fluid (ACSF) containing (mM): choline chloride (110), KCl (2.5), NaH_3_PO_4_ (1.25), myoinositol (3), sodium pyruvate (3), NaHCO_3_ (25), MgCl_2_ (3), CaCl_2_ (0.1); and (μM): TTX (0.1), AP5 (50), DNQX (20).

300-μm sagittal slices were cut on a Leica vibratome. Cerebella were carefully isolated and allowed to recover in carbogenated ACSF at 34°C for 30 minutes. Cerebellar slices were then incubated in carbogenated ACSF containing BxxP (100 μM) at 34°C for 60 minutes. Proximity labeling was initiated by adding 0.3% H_2_O_2_ to the BxxP-ACSF solution at 1:100 (yielding a 0.003% H_2_O_2_ BxxP-ACSF solution); slices were incubated with H_2_O_2_ for 3 minutes. The tissue container was gently swirled to ensure diffusion of H_2_O_2_ throughout the samples. The reaction was then terminated by transferring the tissue to quencher solution, carbogenated ACSF containing freshly added sodium ascorbate (10 mM, Sigma-Aldrich), Trolox (5 mM, Sigma-Aldrich), and NaN_3_ (10 mM, Sigma-Aldrich). Slices were washed in quencher solution five times. Quencher solution was then drained, and slices were collected, snap frozen in liquid nitrogen, and stored at −80°C until further use. At least two slices from each sample submitted for LC-MS/MS-based proteomic analysis were kept for histological validation of labeling efficacy, cell health, and tissue quality; these slices were fixed in 4% paraformaldehyde (PFA) in phosphate-buffered saline (PBS) at 4°C overnight.

Preparation of200–500 μm acute brain slices is a classic standard procedure for electrophysiology experiments and is generally believed to minimize cell and tissue damage while allowing adequate perfusion of tissue by oxygen, reagents, and media (ACSF). Such preparations have been performed using live sagittal cerebellar sections for over 4 decades (Crepel et al., 1981; Llinás and Sugimori, 1980a, 1980b) with few modifications since then. In our study, this has also allowed robust labeling of neuronal cell surfaces in live brain slices (Figures 2C and S1). We anticipate that labeling efficiency may vary across different cell and tissue types but were able to achieve cell-surface labeling in tissue sections ranging from 300 μm to several mm (whole mount/coarse dissection; Figure 1E).

#### Proximity labeling in live non-neural tissues

*UbcCreER^T2^*;*Cre-iPEEL* mice were injected with 4-hydroxytamoxifen (4-OHT) and sacrificed several days later. For heart and fat samples, 4-OHT was injected at P12–14 (50 mg/kg), with sacrifice at P19; for intestine, 4-OHT was injected at P20 (150 mg/kg), with sacrifice at P28; and for kidney and liver, 4-OHT was injected at P36 (50 mg/kg), with sacrifice at P56. 4-OHT was prepared in Chen oil, a 1:4 mixture of castor oil:sunflower seed oil (Sigma-Aldrich, 259853 and S5007) (Guenthner et al., 2013). Expression of the HRP fusion protein did not result in noticeable toxicity to mice or any cell or tissue type we examined. Transgenic expression of plasma membrane-targeted HRP has been performed in a variety of neural and other cell types in diverse model organisms (Hoopfer et al., 2006; Li et al., 2010; Marin et al., 2005; Watts et al., 2004; Zhang et al., 2019) in our laboratory and others without, to our knowledge, any reports of cellular or organismal toxicity.

*UbcCreER^T2^*;*Cre-iPEEL* mice were sacrificed and organs rapidly dissected out in Dulbecco’s Modified Eagle Medium (DMEM) with 10% fetal bovine serum (FBS) and penicillin-streptomycin (P/S) or PBS and sectioned on a vibratome at 300–500 μm (kidney, liver) or processed whole-mount (heart, fat, intestine). Tissue was incubated with BxxP (100 μM) in DMEM-FBS-P/S or PBS for 60 minutes with rotation at room temperature. Proximity labeling was initiated by adding 0.3% H_2_O_2_ to DMEM-FBS-P/S or PBS at 1:100 (yielding a 0.003% H_2_O_2_ solution); tissue was incubated with H_2_O_2_ for 5 minutes. The tissue container was gently swirled to ensure diffusion of H_2_O_2_ throughout the samples. Labeling was terminated by transferring tissue into quencher solution, DMEM-FBS-P/S or PBS containing freshly added sodium ascorbate (10 mM), Trolox (5 mM), and NaN_3_ (10 mM). Tissue was washed in quencher solution five times. Quencher solution was then drained, and tissue was fixed in 4% PFA in PBS at 4°C overnight.

#### Histology

Following overnight fixation in 4% PFA, brain slices and other tissues were washed three times in PBS, then incubated in 10% normal donkey serum (NDS) in PBS with 0.3% Triton X-100 (PBST) for 2–4 hours on a shaker at room temperature. Samples were then incubated in 5% NDS-PBST with rabbit anti-HA antibody (1:500; Cell Signaling Technology) at 4°C for two overnights. Samples were then washed three times in PBST at room temperature and incubated in 5% NDS-PBST with anti-rabbit secondary Cy3-conjugated antibody (1:500; Jackson Immunoresearch) and NeutrAvidin-647 (1:1,000) for two overnights. Samples were then washed once in PBST, incubated in DAPI (Thermo Fisher) in PBST for 30 minutes, and then washed once in PBST and once in PBS before mounting on glass slides in Fluoromount G. Glass coverslips were then mounted on the slides, and slides were incubated at room temperature for at least 4 hours until imaging.

#### Tissue Lysis

Slices were processed in the original collection tube to avoid material loss during transfer. 300 μL high-SDS RIPA buffer [50 mM Tris-HCl (pH 8.0), 150 mM NaCl, 1% sodium dodecyl sulfate (SDS), 0.5% sodium deoxycholate (Sigma-Aldrich), 1% Triton X-100, 1x protease inhibitor cocktail (P8849; Sigma-Aldrich), and 1 mM phenylmethylsulfonyl fluoride (PMSF; Sigma-Aldrich)] was added to each tube of frozen slices. Disposable pestles driven by an electric motor (Thermo Fisher) were used to extensively grind the samples on ice. Lysates of the same experimental group were then merged into a single tube with a final volume of 300 μL high-SDS RIPA buffer. Samples were then vortexed briefly, followed by two rounds of sonication at 4°C (Branson 1800) until the lysate became clear. To denature the postsynaptic density (Loh et al., 2016), samples were heated to 95°C for 5 minutes, then returned onto ice for 1 minute. SDS-free RIPA buffer [50 mM Tris-HCl (pH 8.0), 150 mM NaCl, 0.5% sodium deoxycholate, 1% Triton X-100, 1x protease inhibitor cocktail (P8849), and 1 mM PMSF] was added to each sample to yield 0.2% SDS normal RIPA buffer. Lysates were then rotated at 4°C for 2 hours. Lysates were then transferred to 3.5 mL ultracentrifuge tubes (Beckman Coulter) containing 200 μL of normal RIPA buffer [50 mM Tris-HCl (pH 8.0), 150 mM NaCl, 0.2% SDS, 0.5% sodium deoxycholate, 1% Triton X-100, 1x protease inhibitor cocktail (P8849), and 1 mM PMSF], balanced, and centrifuged at 100,000 g for 30 minutes at 4°C. 3.0 mL of each supernatant was carefully collected and kept on ice. The remaining 200 μL was kept for analysis of the raw lysate by streptavidin blot.

#### Streptavidin enrichment

Streptavidin magnetic beads (Pierce) were used to enrich biotinylated proteins from cerebellar lysates: 150 μL was used with six 300 μm cerebellar slices for biochemistry experiments, and a total of 400 μL was used for each proteomic sample. Calculation of the estimated biotinylated/enriched protein amount based on bead usage and bead binding capacity from the manufacturer (each 100 μL of bead captures an estimated ~55 μg biotinylated rabbit IgG) suggests that we captured ~220 μg proteins per proteomic sample in the labeled/experimental groups. Beads were washed twice with normal RIPA buffer and then incubated with the post-ultracentrifugation lysates on a 4°C rotator overnight (14 hours). Beads were then sequentially washed twice with 1 mL normal RIPA buffer, once with 1 mL 1 M KCl, once with 1 mL 0.1 M Na_2_CO_3_, once with 1 mL 2 M urea in 10 mM Tris-HCl [pH 8.0], and twice with 1 mL normal RIPA buffer. For silver stain or western blot, biotinylated proteins were eluted by heating the beads at 95°C for 10 minutes in 20 μL of 3x protein loading buffer (Bio-Rad) supplemented with 20 mM dithiothreitol (DTT) and 2 mM biotin. For proteomic samples, on-bead trypsin digestion was performed after enrichment (see below for details). All chemicals were purchased from Sigma-Aldrich.

#### Biochemical validation

4%–12% Bis-Tris PAGE gels (Thermo Fisher) were used for protein electrophoresis following the manufacturer’s protocol. A silver stain kit (Pierce) was used for in-gel protein staining. For western blot, proteins were transferred to nitrocellulose membranes (Thermo Fisher). All wash and incubation steps were performed on an orbital shaker at room temperature. After blocking with 3% bovine serum albumin (BSA) in TBST (Tris-buffered saline with 0.1% Tween 20; Thermo Fisher) for 1 hour, membranes were incubated with primary antibodies diluted in 3% BSA in TBST for 1 hour, followed by 4 rounds of 5-minute washes in TBST. Membranes were then incubated with horseradish peroxidase (HRP)-conjugated secondary antibodies diluted in 3% BSA in TBST for 1 hour, followed by 4 rounds of 5-minute washes in TBST. HRP-conjugated streptavidin was used to detect biotinylated protein. Clarity Western ECL blotting substrate (Bio-Rad) and a ChemiDoc imaging system (Bio-Rad) were used for chemiluminescence development and detection.

Primary antibodies used for biochemistry in this study included mouse anti-β-actin (1:2,000; ab8224, Abcam), chicken anti-tubulin (1:1,000; ab89984, Abcam), guinea pig anti-calbindin (1:2,000; Af280, Frontier Institute), rabbit anti-GluD2 (1:2,000; Af500 AB_2571600, Frontier Institute), and mouse anti-mGluR1α (1:2,000; 556331 G209-488, BD Biosciences). HRP-conjugated secondary antibodies (Jackson ImmunoResearch or Thermo Fisher) were used at 1:3,000 – 1:10,000. HRP-conjugated streptavidin (Thermo Fisher) was used at 0.3 μg/mL.

#### On-bead trypsin digestion of biotinylated proteins

The streptavidin-enriched sample (400 μL of streptavidin bead per condition) was processed for on-bead digestion and TMT labeling and used for mass spectrometry analysis. Proteins bound to streptavidin beads were washed twice with 200 μL of 50 mM Tris-HCl buffer (pH 7.5), followed by two washes with 2 M urea/50 mM Tris (pH 7.5) buffer in fresh tubes. The final volume of 2 M urea/50 mM Tris (pH 7.5) buffer was removed, and beads were incubated with 80 μL of 2 M urea/50 mM Tris buffer containing 1 mM dithiothreitol (DTT) and 0.4 μg trypsin. Beads were incubated in the urea/trypsin buffer for 1 hour at 25°C while shaking at 1000 revolutions per minute (rpm). After 1 hour, the supernatant was removed and transferred to a fresh tube. The streptavidin beads were washed twice with 60 μL of 2 M urea/50 mM Tris (pH 7.5) buffer and the washes were combined with the on-bead digest supernatant. The eluate was reduced with 4 mM DTT for 30 minutes at 25°C with shaking at 1000 rpm. The samples were alkylated with 10 mM iodoacetamide and incubated for 45 minutes in the dark at 25°C while shaking at 1000 rpm. An additional 0.5 μg of trypsin was to the sample and the digestion was completed overnight at 25°C with shaking at 700 rpm. After overnight digestion, the sample was acidified (pH < 3) by adding formic acid (FA) such that the sample contained 1% FA. Samples were desalted on C18 StageTips (3M). Briefly, C18 StageTips were conditioned with 100 μL of 100% MeOH, 100 μL of 50% MeCN/0.1% FA, and 2x with 100 μL of 0.1% FA. Acidified peptides were loaded onto the conditioned StageTips, which were subsequently washed 2 times with 100 μL of 0.1% FA. Peptides were eluted from StageTips with 50 μL of 50% MeCN/0.1% FA and dried to completion.

#### TMT labeling and StageTip peptide fractionation

Desalted peptides were labeled with 8 TMT reagents from a 10-plex reagent kit (Thermo Fisher) as directed by the manufacturer. Peptides were reconstituted in 100 μL of 50 mM HEPES. Each 0.8 mg vial of TMT reagent was reconstituted in 41 μL of anhydrous acetonitrile and added to the corresponding peptide sample for 1 hour at room temperature shaking at 1000 rpm. Labeling of samples with TMT reagents was completed with the design described in Figure 3A. TMT labeling reactions were quenched with 8 μL of 5% hydroxylamine at room temperature for 15 minutes with shaking. The entirety of each sample was pooled, evaporated to dryness in a vacuum concentrator, and desalted on C18 StageTips as described above. For the TMT 8-plex cassette, 50% of this sample was analyzed by single-shot LC-MS analysis on a Q-Exactive HF-X MS using the LC-MS/MS methods described below. The other 50% of the sample was fractionated into 6 fractions by basic-reversed phase (bRP) StageTips. A StageTip containing three plugs of SDB material was prepared and conditioned with 100 μL of 100% MeOH, 100 μL of 50% MeCN/0.1% FA, and 2x with 100 μL of 0.1% FA. The sample was resuspended in 200 uL 0.1% FA (pH < 3) and loaded onto the conditioned StageTip and eluted in a series of buffers with increasing MeCN concentrations. Six fractions were collected in 20 mM ammonium formate (5%, 10%, 15%, 20%, 25%, and 45% MeCN), dried to completion and analyzed by LC-MS/MS on a Q-Exactive Plus MS using the LC-MS/MS methods described below.

#### Liquid chromatography-tandem mass spectrometry

Desalted, TMT-labeled peptides were resuspended in 9 μL of 3% MeCN, 0.1% FA and analyzed by online nanoflow liquid chromatography tandem mass spectrometry (LC-MS/MS) using a Q Exactive HF-X (for single-shot analysis) or Q Exactive Plus (for fractionated samples) (Thermo Fisher) coupled online to a Proxeon Easy-nLC 1200 (Thermo Fisher). 4 μL of each sample were loaded at 500 nL/min onto a microcapillary column (360 μm outer diameter x 75 μm inner diameter) containing an integrated electrospray emitter tip (10 mm), packed to approximately 28 cm with ReproSil-Pur C18-AQ 1.9 mm beads (Dr. Maisch GmbH) and heated to 50°C. The HPLC solvent A was 3% MeCN, 0.1% FA, and the solvent B was 90% MeCN, 0.1% FA. Peptides were eluted into the mass spectrometer at a flow rate of 200 nL/min. The single-shot sample on Q Exactive HF-X was analyzed using a 260 min LC-MS/MS method with the following gradient profile: (min:%B) 0:2; 1:6; 235:30; 244:60; 245:90; 250:90; 251:50; 260:50 (the last two steps at 500 nL/min flow rate). The Q Exactive HF-X was operated in the data-dependent mode acquiring HCD MS/MS scans (r = 45,000) after each MS1 scan (r = 60,000) on the top 20 most abundant ions using an MS1 target of 3E6 and an MS2 target of 1E5. The maximum ion time utilized for MS/MS scans was 120 ms; the HCD normalized collision energy was set to 31; the dynamic exclusion time was set to 20 s, and the peptide match and isotope exclusion functions were enabled. Charge exclusion was enabled for charge states that were unassigned, 1 and > 7. The fractionated samples on Q Exactive Plus were run with 110-minute method, which used the following gradient profile: (min:%B) 0:2; 1:6; 85:30; 94:60; 95:90;100:90; 101:50; 110:50 (the last two steps at 500 nL/min flow rate). The Q Exactive Plus was operated in the data-dependent mode acquiring HCD MS/MS scans (r = 35,000) after each MS1 scan (r = 70,000) on the top 12 most abundant ions using an MS1 target of 3E6 and an MS2 target of 5E4. The maximum ion time utilized for MS/MS scans was 120 ms; the HCD normalized collision energy was set to 30; the dynamic exclusion time was set to 20 s, and the peptide match was set to “Preferred” and isotope exclusion functions were enabled. Charge exclusion was enabled for charge states that were unassigned, 1 and > 7.

#### Mass spectrometry data processing

Collected data were analyzed using the Spectrum Mill software package v6.1 pre-release (Agilent Technologies). Nearby MS scans with the similar precursor m/z were merged if they were within ±60 s retention time and ±1.4 m/z tolerance. MS/MS spectra were excluded from searching if they failed the quality filter by not having a sequence tag length 0 or did not have a precursor MH+ in the range of 750–4000. All extracted spectra were searched against a UniProt database containing mouse reference proteome sequences. Search parameters included: ESI QEXACTIVE-HCD-v2 scoring parent and fragment mass tolerance of 20 ppm, 40% minimum matched peak intensity, trypsin allow P enzyme specificity with up to two missed cleavages, and calculate reversed database scores enabled. Fixed modifications were carbamidomethylation at cysteine. TMT labeling was required at lysine, but peptide N termini were allowed to be either labeled or unlabeled. Allowed variable modifications were protein N-terminal acetylation and oxidized methionine. Individual spectra were automatically assigned a confidence score using the Spectrum Mill auto-validation module. Score at the peptide mode was based on target-decoy false discovery rate (FDR) of 1%. Protein polishing auto-validation was then applied using an auto thresholding strategy. Relative abundances of proteins were determined using TMT reporter ion intensity ratios from each MS/MS spectrum and the median ratio was calculated from all MS/MS spectra contributing to a protein subgroup. Proteins identified by 2 or more distinct peptides and ratio counts were considered for the dataset.

#### Proteomic data analysis

To determine the cutoff in each biological replicate, we applied ratiometric analysis (Hung et al., 2014; Li et al., 2020). Detected proteins were classified according to the annotation of subcellular localization in the UniProt database (retrieved in Apr 2020). Proteins with the plasma membrane annotation were classified as true-positives (TPs). Proteins with either nuclear, mitochondrial, or cytoplasmic annotations but without the plasma membrane annotation were classified as false-positives (FPs). Of the total of 4752 detected proteins, 819 were TPs and 2228 were FPs. For each replicate, proteins were first ranked in descending order according to TMT ratio (129C/127C, 128C/127N, 130N/128N, 129N/126). For each protein on each ranked list, the accumulated true- and false-positive counts above its TMT ratio were calculated. A receiver operating characteristic (ROC) curve was plotted for each replicate. The cutoff was set where the *true-positive rate minus false-positive rate* (TPR – FPR) was maximized: 129C/127C: 0.2672, 128C/127N: 0.2549, 130N/128N: 0.3351, 129N/126: 0.3008. Post-cutoff proteomic lists of the two biological replicates for each time point were intersected to obtain the final proteomes. We also performed cutoff analyses with a different TMT pairing regime (129C/127N, 128C/127C, 130N/126, 129N/128N) and obtained almost identical proteomes. Alternative cutoff methods with more stringent inclusion criteria (requiring proteins to have higher experimental-to-control TMT ratios than the cutoff thresholds in all four possible ratiometric combinations) produced smaller proteomes with similar gene ontology characteristics (Figures S2B-S2D).

For gene ontology analysis, we uploaded each proteome to the STRING database search portal and plotted the top five or ten “cellular compartment” and “biological process” retrieved terms with the lowest false discovery rates.

#### Dendrite morphogenesis candidate screen

Standard cloning procedures were used to generate new DNA constructs. Plasmids constructs encoding Cas9 and guide RNAs each had two guide RNAs subcloned into the *pX333* plasmid vector (Addgene 64073; see Table S2 for gRNA sequences). These constructs were co-electroporated with *pCAG-eGFP* plasmid (Addgene 11150). Loss-of-function microRNA (miR) plasmid constructs encoded pCAG-driven Emerald GFP (EmGFP) followed by a single miR (see Table S2 for miR sequences); the miR plasmid backbone was a generous gift from M. Yuzaki. Armh4 overexpression plasmid constructs were subcloned into a pCAG vector and had two hemagglutinin (HA) tags separated by GSG linkers and Armh4 coding DNA sequences: Armh4^WT^ and Armh4^ΔICD^ had 2xHA located immediately after the signal peptide while Armh4^Endo6A^ had 2xHA located immediately before the stop codon. The Armh4^WT^ constructs used in costains with 1) Rab7 had 2xHA tags located immediately before the stop codon; 2) Lamp1 had 2xHA tags located immediately after the signal peptide; and 3) Rab3 had a V5 tag located immediately after the signal peptide. The Armh4^ΔICD^ construct had all intracellular amino acids beginning with K737 deleted and replaced with an inert GSG linker followed directly by a stop codon. The Armh4^Endo6A^ construct had the intracellular amino acid sequence DRVMLL mutated to AAAAAA.

Plasmid DNA for in utero electroporation (IUE) was purified using Qiagen plasmid maxiprep kits (Qiagen) and, following ethanol precipitation, dissolved in HEPES-buffered saline. Plasmid solutions were colored with 0.01% Fast Green so as to be visible when injected into the fourth ventricle. The plasmid DNA concentrations used for IUE were as follows: 2 μg/μL for *pCAG-EmGFP-Armh4-miR*; or 1 and 2 μg/μL for *pCAG-GFP* and *pCAG-2xHA-Armh4^WT^/pCAG-2xHA-Armh4*^Δ*ICD*^*/pCAG-Armh4^Endo6A^-2xHA* or *pX333-LacZ/Armh4*, respectively.

LacZ-gR1 target sequence: TGCGAATACGCCCACGCGAT

LacZ-gR2 target sequence: CGGCGCGTAAAAATGCGCTC

LacZ-miR target sequence: AAATCGCTGATTTGTGTAGTC

Armh4-gR1 target sequence: GAGCACTACCACCAAGTATT

Armh4-gR2 target sequence: GCTCCAATGGTACTATCTGA

Armh4-miR target sequence: TATGAGCAGACCAACTCTGAT

Other gR and miR sequences used in the screen are listed in Table S2.

IUE was performed as described elsewhere (Nishiyama et al., 2012; Takeo et al., 2021). Wild-type CD1 pregnant dams (Charles River) were anesthetized at 11.5 days post-conception (E11.5) with isoflurane (starting at 2.5% and maintained at 1.5% L O_2_/min). After cleaning the abdomen with betadine, a laparotomy was performed, uterine horns were exposed, and DNA was injected within the following 20–30 minutes. To relax the myometrium, ritodrine hydrochloride (0.4–0.8 μg/g; Sigma-Aldrich) was injected into the abdominal cavity or directly onto the exposed uterine horns. Warm sterile PBS was continually applied to the embryos to hydrate them. Under the illumination of a fiber optic light source (Dolan Jenner) with a flexible light guide (Allied Electronics), plasmid DNA solution in a glass capillary needle was injected into the fourth ventricle using a microinjector (Eppendorf FemtoJet 4I; Eppendorf) until the rostral region of the fourth ventricle was filled with DNA, as visualized with Fast Green dye (Sigma), and 2–3 μL was injected into each embryo. After injection, the embryo was held through the uterus with tweezer-style electrodes (CUY650P3; NEPAGENE) so that the positive metal electrode was placed on the rostral rhombic lip of the fourth ventricle, and 1–4 sets of electrical pulses (five pulses of 33–38 V, each with a duration of 30 ms, at intervals of 970 ms) were delivered by an electroporator (ECM 399, BTX). After electroporation, the uterus was returned to the abdominal cavity and 0.05–0.10 mg/kg buprenorphine-SR was injected directly into the intraperitoneal space. The abdominal wall and skin were then sutured closed. The dams were kept on a heating pad until recovery from anesthesia, then returned to their home cages. Embryos were allowed to continue developing and were typically born ~E19. After birth, pups were screened for successful electroporation by examining their cerebella through the skin and skull under a fluorescence stereomicroscope, then returned to their home cage with the dam.

#### Tissue processing for dendrite morphology analysis

Mice were euthanized with Avertin and perfused with 10 mL PBS and 10–25 mL 4% PFA in PBS. Brains were dissected out and postfixed in 4% PFA overnight (12–24 hours) at 4°C and stored in PBS at 4°C until further processing. Then 100 μm sagittal cerebellar sections were collected on a vibratome, washed twice in PBS, and incubated in 10% normal donkey serum (NDS) in PBST for 2 hours on a shaker at room temperature. For anti-Rab/Lamp1 stains, 50 μm sections were heated in a microwave for 1 minute in citrate acid buffer in H_2_O (pH 6.0; 8.2 mM Na_3_C_6_H_5_O_7_, Mallinckrodt Chemicals; 1.8 mM C_6_H_8_O_7_, Sigma Aldrich) for heat-mediated antigen retrieval before washing and blocking. Sections were then incubated in 5% NDS-PBST with primary antibodies at 4°C for 36–48 hours: rabbit anti-Armh4 (1:600; Millipore Sigma), rabbit anti-HA (1:500; Cell Signaling Technology), mouse anti-HA (1:1,000; Cell Signaling Technology), mouse anti-V5 (1:1,000; ThermoFisher), rabbit anti-Rab3 (1:100; ProteinTech), rabbit anti-Rab7 (1:100; Abcam), goat anti-tdTomato (1:1,000; Origene), rabbit anti-Lamp1 (1:100; Abcam). Slices were then washed three times in PBST and incubated in 5% NDS-PBST with fluorophore-conjugated secondary antibodies (1:500; Jackson ImmunoResearch) for 2 hours at room temperature. Slices were then washed once in PBST, incubated in DAPI (Sigma-Aldrich, 1:10,000 in PBST) for 30 minutes, then washed once in PBST and once in PBS before mounting on Superfrost Plus glass slides (Fisher Scientific) in Fluoromount-G (SouthernBiotech). Glass coverslips were then mounted on the slides, and slides were incubated at room temperature for at least 4 hours until imaging.

#### Tissue processing for RNAscope in situ hybridization

A postnatal day 14 mouse was deeply anesthetized via intraperitoneal Avertin injection and decapitated into 0.05 M PBS. The brain was immediately dissected out, immersed in optimal cutting temperature media (Tissue-Tek), and frozen in liquid nitrogen. The frozen brain was stored at −80°C in an air-tight bag until used. A cryostat was used to collect 10-μm sagittal brain sections. In situ hybridization using RNAScope Multiplex Fluorescent Kit v.2 (ACD Bio) was performed within 2 days of sectioning. Armh4 mRNA was detected using probe-Armh4-C1 (ACD Bio, 1085041-C1) following the manufacturer’s protocols. The sample was counterstained with DAPI and mounted in ProLong Gold Antifade Mountant (ACD Bio).

#### Confocal image acquisition

Brightly fluorescent Purkinje cells with intact dendritic arbors within the flat banks of cerebellar lobules 2–9 were imaged. Labeled cells with arbors interdigitating with other labeled cells’ arbors were avoided. Images were acquired on a Zeiss LSM 780 laser-scanning confocal microscope (Carl Zeiss), with a 40x/1.4 Plan-Apochromat oil objective (Carl Zeiss). Confocal z stacks for fine morphological analysis were obtained at 0.36–0.44 μm intervals with an x-y resolution of 2048×2048 pixels. Dendritic arbors were traced using Imaris 9.3 FilamentTracer (Oxford Instruments).

### QUANTIFICATION AND STATISTICAL ANALYSIS

Statistical tests and numbers of independent replicates per experiment are indicated in figure legends. No statistical methods were used to determine sample sizes. Data collection and analysis were not performed blind to the conditions of the experiments. Excel (Microsoft) and Prism (GraphPad) were used for data analysis and plotting.

#### Quantitative comparison of developing and mature proteomes

For the volcano plot (Figure 4D) comparing differentially enriched proteins in developing and mature samples, a linear model was fit to account for the variance across replicates for each stage and normalize data by the appropriate negative control samples. A protein summary was first generated wherein each TMT condition was calculated as a ratio to the median intensity of all the channels, enabling all channels to have the same denominator. Then, for each protein, a linear model was used to calculate the following ratios and corresponding *p-values*:

$$\frac{mature\;labeling\;condition\;(130N,129N)-mature\;negative\;controls\;(128N,126)}{developing\;labeling\;conditions\;(129C,128C)-developing\;negative\;controls\;(127C,127N)}$$

Using log_2_ transformed TMT ratios, the linear model is as follows: log_2_(TMT ratio)~MATURE*TRT, where MATURE and TRT (treatment) are indicator variables representing maturity (MATURE = 1 for mature, 0 for developing) and labeling condition (TRT = 1 for labeled, 0 for negative control), respectively. Including an interaction term yields: log_2_(TMT ratio) = b_0_ + b_1_ MATURE + b_2_ TRT + b_3_ [MATURE×TRT], where the b_3_ coefficient represents the required (log-transformed) ratio between mature and developing conditions taking into account the appropriate negative controls and replicates. A moderated t-test was used to test the null hypothesis b_3_ = 0 and calculate nominal *p-values* for all proteins. These nominal *p-values* were then corrected for multiple testing using the Benjamini-Hochberg FDR (BH-FDR) method (Benjamini and Hochberg, 1995). The linear model along with the associated moderated t-test and BH-FDR correction were implemented using the limma library (Ritchie et al., 2015) in R.

Note that ratio compression is an inherent technical limitation of current state-of-the-art multiplexed quantitative proteomics based on MS/MS, as performed in this study. For example, if one spikes two exogenous proteins at a 2:1 ratio into a lysate sample, the TMT/iTRAQ ratio resulting from MS will always be much smaller than 2.0. This is due to imperfect MS1 precursor ion selection and coeluting peptide, such that when MS2 TMT fragments are quantified, the ratio will always move toward 1.0, the median of the sample. This is explained in great detail in a number of classic proteomic papers and is widely acknowledged in the proteomics field (Hogrebe et al., 2018; Karp et al., 2010; Savitski et al., 2013). Thus, fold-changes in our data actually represent larger protein level fold-changes.

While ratio compression can compromise the accuracy of quantification of TMT-labeled peptides (Savitski et al., 2013; Ting et al., 2011), it is generally not possible to estimate the amount of compression without spiking in standard proteins. Synchronous precursor selection triple-stage mass spectrometry (SPS-MS3) (Ting et al., 2011) reduces compression and improves quantitative accuracy but is accompanied by a loss of up to 30% in peptide identification. Compression increases with sample complexity and is greatly reduced when analyzing less complex samples or when samples are fractionated offline to reduce complexity prior to MS. Our samples were less complex than entire cellular proteomes and, additionally, were fractionated offline prior to MS. Therefore, we expect that the compression in our sample is less than that of entire cellular proteomes.

Although MS/MS-based quantification has this ratio compression effect, resulting in inaccurate quantification, it is the most precise measurement method possible with current MS methods (Hogrebe et al., 2018), compared to label-free quantification, SILAC (MS1), or MS3. This precision means that the ratio is consistent and not easily affected by background or technical bias, such that small differences or changes in TMT ratios can be interpreted with confidence to represent bona fide biological changes, even though ratios are compressed to be smaller.

#### Comparison of transcriptomics and proteomics

We focused comparison of protein abundance in our cell-surface proteomes to levels of corresponding RNAs detected in transcriptomic studies on the top 100 CSPs identified in our P35 cell-surface Purkinje cell proteome (130N/128N; Table S1B). We used the rank order of proteins enriched in this dataset as a proxy for CSP abundance and used corresponding RNA expression levels from two studies, one using single-nucleus RNA sequencing (snRNAseq) of cerebellar cells (Kozareva et al., 2021) and the other translating ribosomal affinity purification (TRAP) of Purkinje cells followed by microarray analysis (Buchholz et al., 2020). In each case, we utilized adult/mature timepoint data. We used the Broad Single Cell portal (https://singlecell.broadinstitute.org/single_cell/study/SCP795/a-transcriptomic-atlas-of-the-mouse-cerebellum) to construct a dot plot of RNA expression levels in Purkinje cells and neighboring cell types from the snRNAseq data (Figures S3A and S3B) (Kozareva et al., 2021). We used a supplementary file of the TRAP data (Buchholz et al., 2020) to generate a heatmap of translating mRNA expression levels by averaging replicate P56 mRNA expression values, subtracting the microarray background intensity, and taking the anti-log_2_ (Figure S3A).

#### Quantification of Purkinje cell dendrite morphology and related parameters

Low-quality images (either due to dim GFP fluorescence or interdigitating labeled dendritic arbors) were analyzed for height deficiency and number of primary dendrites but were not traced and so did not contribute to quantifications of total dendrite length and number of branchpoints. This accounts for the small discrepancy in *n* between these different phenotypic measures.

Images were acquired to maximize the dynamic range of fluorescence, such that at most only a few pixels in any single image plane were saturated. To correlate GFP expression level (a proxy for miR expression level) with phenotypic severity/measures, we analyzed images in which a very bright *Armh4-miR* cell (all of which had very strong phenotypes) was in the same field-of-view as one or more dimmer *Armh4-miR* cells. Mean GFP fluorescence was measured at the cell body of z-stacked images using Fiji (ImageJ). The brightest cells had mean somal GFP fluorescence levels of 218–249 (255 representing saturation), and dimmer cells had somal GFP fluorescence levels ranging from 70 to 192 (representing lower GFP and thus miR expression levels). These quantifications thus allowed us to address whether miR expression levels correlated with phenotypic severity.

Imaris 9.3 FilamentTracer (Oxford Instruments) was used to trace the dendrites of cerebellar Purkinje cells from z stack confocal images. Dendrites were traced using semi-automatic AutoPath and Manual modes with a fixed dendrite diameter of 5 pixels. The dendrite beginning point was defined as the location where the primary dendrite thickness was 8 μm in diameter. All dendritic protrusions longer than 2 μm were traced. After tracing all dendrites, total dendritic length and total number of dendritic branch points were obtained via the Statistics function. Images of cells and traces (“filament” objects) were obtained using the Snapshot function; traces were collected at 7 pixels.

For analysis of vGluT1 puncta, vGluT1 signal was thresholded using Fiji’s “Threshold” function; the freehand selection tool was used to outline the extent of the LOF cell’s dendritic arbor in each image plane; the “Measure” function was used to determine the size of the area of interest, and puncta therein were quantified using the “Analyze Particles” function, with puncta size set at 0.25–2 μm^2^ to limit measurements to large vGluT1 + puncta. Nearby regions in the molecular layer of the same image plane were used as controls and for normalization. All control and LOF regions were in the deepest third of the molecular layer, since Armh4 LOF cells rarely arborize much more superficially.

## Supplementary Material

1 Table S1. Raw and processed proteome data, related to Figure 3 (see Excel sheets)
(A) P15 Purkinje cell-surface proteome by the cutoff shown in Figures 3F and 3G. Proteins are ranked by the TMT ratio 129C:127C in descending order.
(B) P35 Purkinje cell-surface proteome by the cutoff shown in Figures 3F and 3G. Proteins are ranked by the TMT ratio 130N:128N in descending order.
(C) P15 Purkinje cell-surface proteome by the cutoff shown in Figure S2B. Proteins are ranked by the TMT ratio 129C:127C in descending order.
(D) P35 Purkinje cell-surface proteome by the cutoff shown in Figure S2B. Proteins are ranked by the TMT ratio 130N:128N in descending order.
(E) P15 Purkinje cell-surface proteome by the cutoff shown in Figure S2C. Proteins are ranked by the TMT ratio 129C:127C in descending order.
(F) P35 Purkinje cell-surface proteome by the cutoff shown in Figure S2C. Proteins are ranked by the TMT ratio 130N:128N in descending order.
(G) P15 Purkinje cell-surface proteome by the cutoff shown in Figure S2D. Proteins are ranked by the TMT ratio 129C:127C in descending order.
(H) P35 Purkinje cell-surface proteome by the cutoff shown in Figure S2D. Proteins are ranked by the TMT ratio 130N:128N in descending order.
(I) Protein expression changes between P15 and P35 samples and the corresponding *p*-values. Proteins are ranked by *p*-value in ascending order. Note that this list includes all detected proteins prior to cutoff analysis and thus contains intracellular contaminants.
(J) TMT ratios of all detected proteins prior to cutoff analysis.
(K–R) Each tab shows one experimental-to-control TMT ratio (see Figure 3A for detail). All detected proteins prior to cutoff analysis are listed and ranked by the corresponding TMT ratio in descending order.

2

## Figures and Tables

**Table:** KEY RESOURCES TABLE

REAGENT or RESOURCE	SOURCE	IDENTIFIER
Antibodies		
rabbit anti-HA	Cell Signaling Technology	Cat# 3724; RRID: AB_1549585
mouse anti-β-actin	Abcam	Cat# ab8224; RRID: AB_449644
chicken anti-tubulin	Abcam	Cat# ab89984; RRID: AB_10672056
guinea pig anti-calbindin	Frontier Institute	Cat# Af280; RRID: AB_2571570
rabbit anti-GluD2	Frontier Institute	Cat# Af500; RRID: AB_2571600
mouse anti-mGluR1α	BD Biosciences	Cat# 556331; RRID: AB_396369
rabbit anti-Armh4	Millipore Sigma	Cat# HPA001789; RRID: AB_1078328
mouse anti-HA	Cell Signaling Technology	Cat# 2367; RRID: AB_10691311
mouse anti-V5	ThermoFisher	Cat# R960-25; RRID: AB_2556564
rabbit anti-Rab3	ProteinTech	Cat# 15029-1-AP; RRID: AB_2177378
rabbit anti-Rab7	Abcam	Cat# EPR7589/ab137029; RRID: AB_2629474
goat anti-tdTomato	Origene	Cat# AB8181-200; RRID: AB_2722750
rabbit anti-Lamp1	Abcam	Cat# EPR21026/ab208943
Alexa Fluor 488 donkey anti-rabbit IgG antibody	Jackson ImmunoResearch Labs	Cat# 711-545-152; RRID: AB_2313584
Cy3 donkey anti-rabbit IgG antibody	Jackson ImmunoResearch Labs	Cat# 711-165-152; RRID: AB_2307443
Cy3 donkey anti-mouse IgG antibody	Jackson ImmunoResearch Labs	Cat# 715-165-151; RRID: AB_2315777
Alexa Fluor 647 mouse anti-rabbit IgG antibody	Jackson ImmunoResearch Labs	Cat# 715-605-150; RRID: AB_2340862
Alexa Fluor 647 rabbit anti-rabbit IgG antibody	ThermoFisher Scientific	Cat# A-31573; RRID: AB_2536183
Bacterial and Virus Strains		
Biological Samples		
Chemicals, Peptides, and Recombinant Proteins		
Isoflurane	Henry Schein Animal Health	CAS# 26675-46-7; CHEBI:6015
Avertin (2,2,2-Tribromoethanol)	Sigma	SKU# T48402
DAPI	ThermoFisher Scientific	Cat# D1306
Fast Green dye	Millipore Sigma	Cat# F7258
Buprenorphine-SR	ZooPharm	lot # BSRLAB0.5-191112
Ritodrine hydrochloride	Sigma	R0758
Triton X-100	Millipore Sigma	T8787
Fluoromount-G	ThermoFisher Scientific	Cat# 00-4958-02
normal donkey serum	Jackson ImmunoResearch Labs	Cat# 017-000-121; RRID:AB_2337258
Critical Commercial Assays		
Mass spectrometry proteomics data	This paper	MassIVE: MSV000088506
RNA sequencing data: Purkinje cell TRAP	Buchholz et al., 2020	NCBI Gene Expression Omnibus: GSE140307; https://www.pnas.org/doi/full/10.1073/pnas.2000102117#supplementary-materials
RNA sequencing data: cerebellum snRNAseq	Broad Institute Single Cell Portal; Kozareva et al., 2021	NCBI Gene Expression Omnibus: GSE165371; https://singlecell.broadinstitute.org/single_cell/study/SCP795/
Experimental Models: Cell Lines		
Experimental Models: Organisms/Strains		
Mouse: Crl:CD1(ICR)	Charles River	RRID:IMSR_CRL:022
Mouse: Pcp2-Cre	JAX, Zhang et al., 2004	JAX stock #010536; RRID:IMSR_JAX:010536
Mouse: Flp deleter	JAX, Farley et al., 2000	JAX stock #009086; RRID:IMSR_JAX:009086
Mouse: Ubc-CreERT2	JAX, Ruzankina et al., 2007	JAX stock #007001; RRID:IMSR_JAX:007001
Mouse: dual-iPEEL	This paper	N/A
Mouse: Cre-iPEEL	This paper	N/A
Mouse: Cd47^KO^	JAX, Lindberg et al., 1996	JAX stock #003173; RRID:IMSR_JAX:003173
Mouse: MADM16-GT	Contreras et al., 2021	N/A
Mouse: MADM16-TG	Contreras et al., 2021	N/A
Mouse: Thsd7a^KO^	Gift from S.B. Nelson; Clark et al., 2020	N/A
Mouse: MADM6-GT	Contreras et al., 2021	N/A
Mouse: MADM6-TG	Contreras et al., 2021	N/A
Oligonucleotides: see Table S2 for a complete list of sequences.		
Recombinant DNA: see Table S2 for a complete list of gR and miR sequences integrated into plasmids.		
pCAG-GFP	Matsuda and Cepko, 2004	RRID: Addgene_11150
pCAG-tdTomato	W. Joo, unpublished	N/A
pX333	Maddalo et al., 2014	RRID:Addgene_64073
pX333-CBh-Cas9-U6-2xsgRNA-LacZ	Takeo et al., 2021	N/A
pX333-CBh-Cas9-U6-2xsgRNA-Armh4	This paper	N/A
pCAG-EmGFP-miR	Gift from M. Yuzaki	N/A
pCAG-EmGFP-LacZ-miR	This paper	N/A
pCAG-EmGFP-Armh4-miR	This paper	N/A
pCAG-2xHA-Armh4^WT^	This paper	N/A
pCAG-V5-Armh4^WT^	This paper	N/A
pCAG-Armh4^WT^-2xHA	This paper	N/A
pCAG-2xHA-Armh4^ΔICD^	This paper	N/A
pCAG-Armh4^Endo6A^-2xHA	This paper	N/A
Software and Algorithms		
ZEN	Carl Zeiss	RRID: SCR_013672
Imaris 9.3	Oxford Instruments	RRID:SCR_007370; https://imaris.oxinst.com/
ImageJ (Fiji)	NIH	https://imagej.net/Fiji/Downloads
Prism 9	GraphPad	RRID:SCR_002798; https://www.graphpad.com/
Excel	Microsoft	RRID:SCR_016137
Spectrum Mill	Agilent	https://proteomics.broadinstitute.org/millhome.htm

## Highlights

- *iPEEL*: a method for cell-type-specific in situ cell-surface proteomics in mice
- *iPEEL* labels cell-surface proteomes of a variety of cell types from diverse organs
- Cell-surface proteomes of developing and mature cerebellar Purkinje cells profiled
- Armh4, a proteome-informed candidate, has a critical role in dendrite morphogenesis
