# Supplementary material for: In situ cell-type-specific cell-surface proteomic profiling in mice
Source: Neuron. Author manuscript; Available in PMC 2023 Dec 7. (PMC9742329; doi:10.1016/j.neuron.2022.09.025)
Supplement: 2 [file NIHMS1839240-supplement-2.pdf]

Supplementary Information

In situ cell-type-specific cell-surface proteomic profiling in mice

S. Andrew Shuster, Jiefu Li, URee Chon, Miley C. Sinantha-Hu, David J. Luginbuhl, Namrata D. Udeshi, Dominique Kiki Carey, Yukari H. Takeo, Qijing Xie, Chuanyun Xu, D.R. Mani, Shuo Han, Alice Y. Ting, Steven A. Carr, Liqun Luo

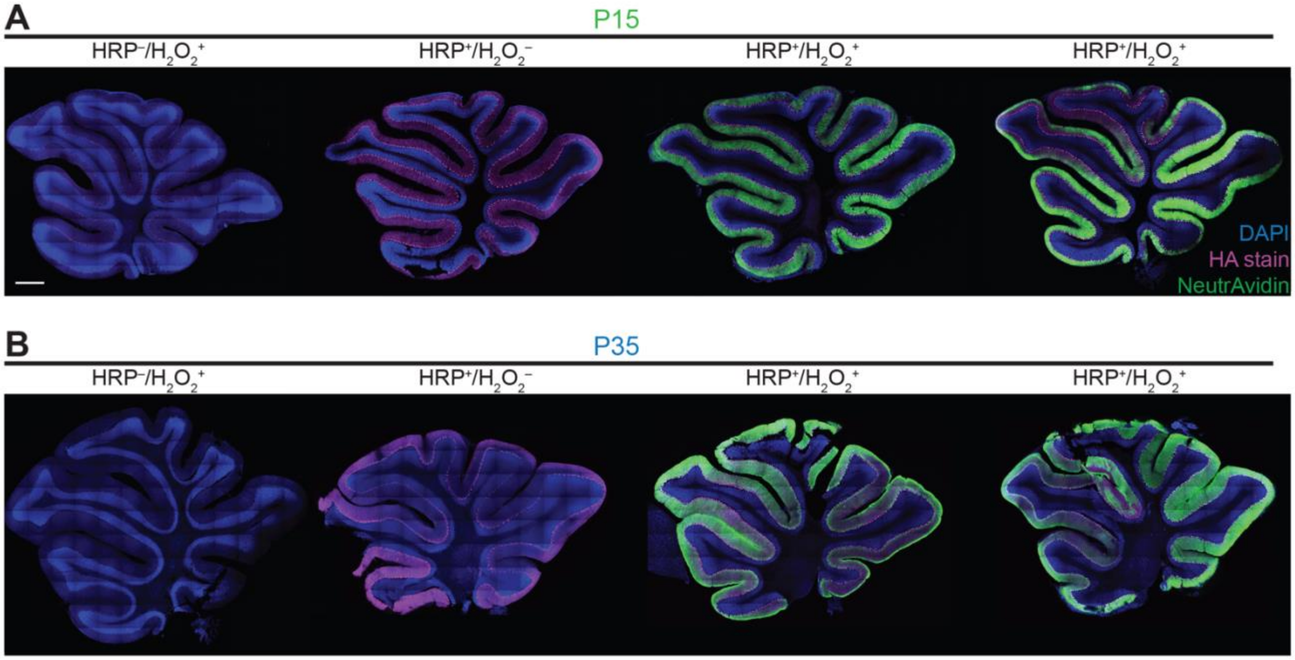

Figure S1. Histological evaluation of cerebellar Purkinje cell-surface biotinylation by iPEEL, related to Figure 2

Example images of P15 (A) and P35 (B) whole cerebellum samples from negative control ($HRP^-/H_2O_2^+$, $HRP^+/H_2O_2^-$) and two experimental replica ($HRP^+/H_2O_2^+$) conditions (Figure 3A), showing consistent cerebellum-wide selective labeling of the Purkinje cell surface. All images are of 300-µm sagittal sections. A 300-µm slice from each cerebellum that went into a proteomic sample (Figure 3A) was saved for histological validation of tissue health (indicated by the absence of abnormal cell morphology or blebbing), widespread cell-surface labeling, and a general absence of intracellular labeling. Four cerebella were combined for each P15 proteomic sample; one cerebellum was used for each P35 proteomic sample. Scale bar, 500 µm.

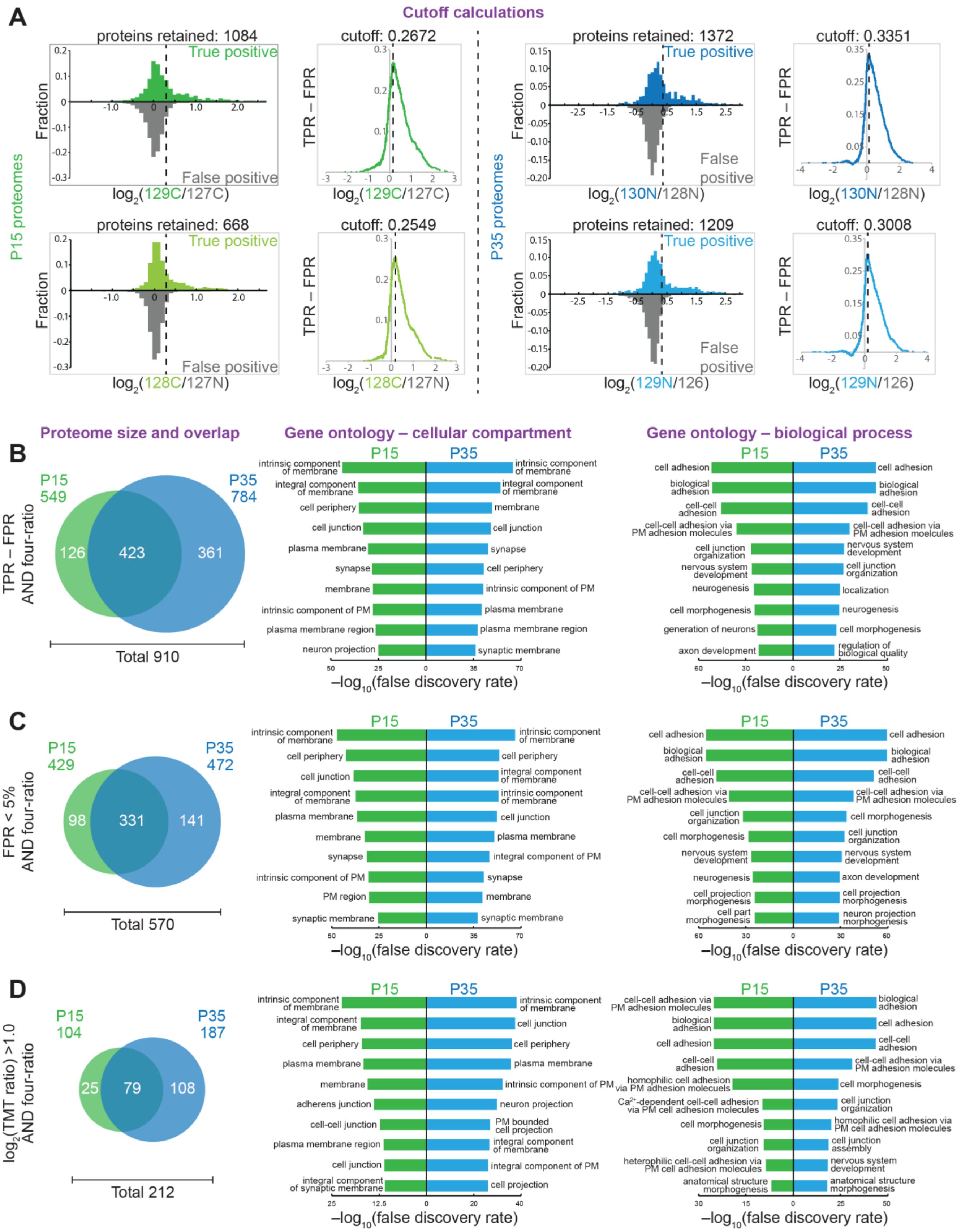

Figure S2. Cutoff and additional proteomic analysis, related to Figures 3 and 4

(A) Determination of the tandem mass tag (TMT) ratio cutoff in each biological replicate. Cutoffs were set where *true-positive rate – false-positive rate* (TPR – FPR) maximized. True-positive denotes proteins with plasma membrane annotation by the UniProt database. False-positive includes nuclear, mitochondrial, and cytosolic proteins without plasma membrane annotation by UniProt.
(B–D) In these analyses, a protein must have higher experimental-to-control TMT ratios than the cutoff thresholds in all four possible ratiometric combinations (P15: 129C/127C, 129C/127N, 128C/127C, and 128C/127N; P35: 130N/128N, 130N/126, 129N/128N, and 129N/126) to be included in the final proteome. Three different cutoff methods yielded proteomes of various sizes but almost identical gene ontology features: (B) cutoff at the maxima of [*true-positive rate – false-positive rate* (TPR – FPR)]; (C) false-positive rate (FPR) < 5%; and (D) $\log_2$(experimental-to-control TMT ratio) > 1.0. Note that the cutoff approach in (D) relies solely on TMT ratios and not protein annotation. PM, plasma membrane.

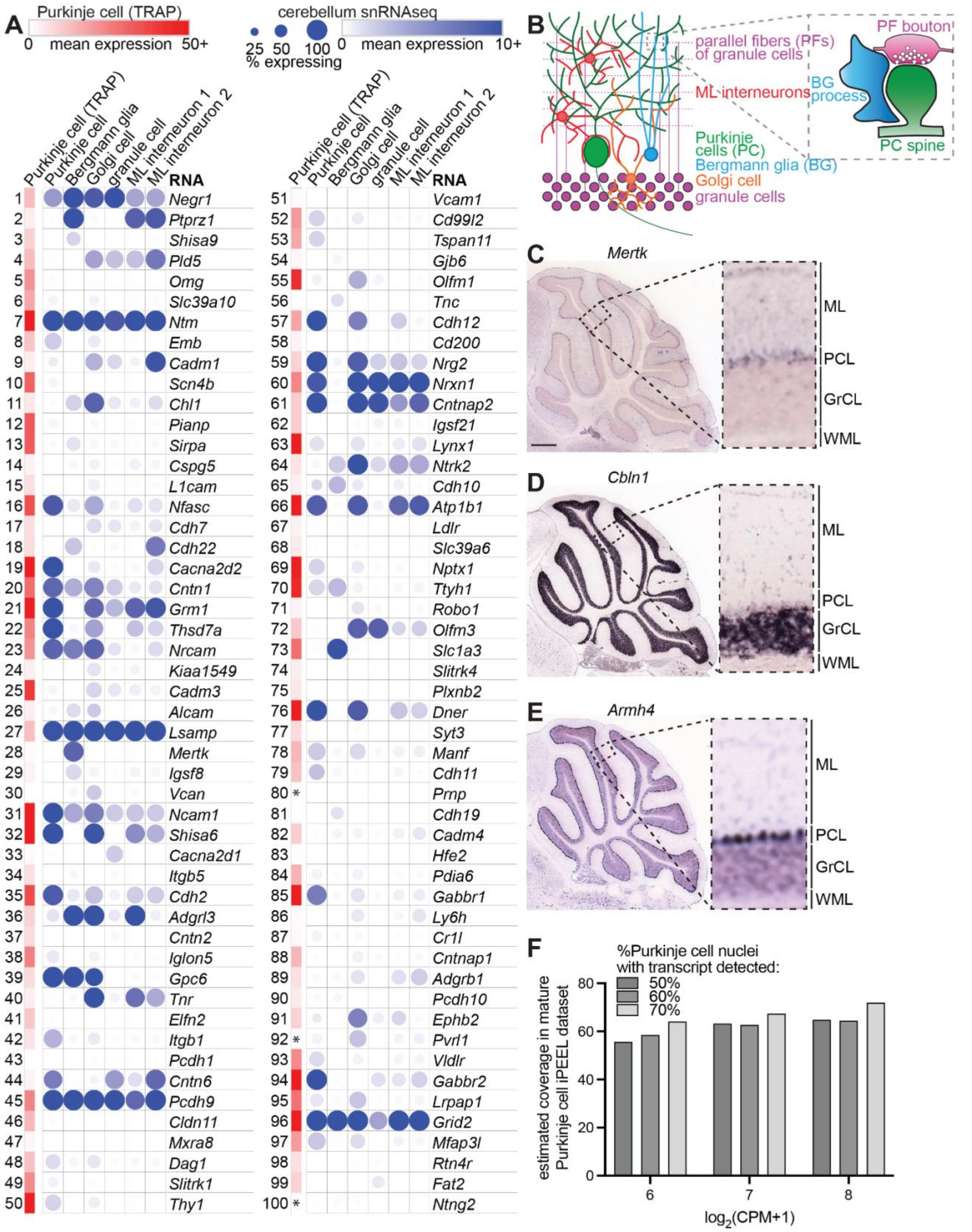

Figure S3. Comparison of proteins from mature Purkinje cell-surface proteome and RNAs from transcriptome profiling in adult cerebellum, related to Figure 4

(A) Comparison of the top 100 most enriched P35 Purkinje cell CSPs with RNAs detected by translating ribosomal affinity purification (TRAP) in Purkinje cells followed by microarray analysis (Buchholz et al., 2020) (column 1) and single-nucleus RNA sequencing (snRNAseq) of Purkinje cells and other cerebellar cell types in close contact with Purkinje cells (Kozareva et al., 2021) (columns 2–7). Scales for expression levels and percentage of expressing cells (snRNAseq) are indicated. RNAs are listed by protein enrichment rank in the P35 Purkinje cell-surface proteome (130N/128N; see Figure 3A and Table S1) from 1 (highest) to 100 (lowest). Asterisks (*) represent mRNAs absent from the TRAP dataset. As is evident from this comparison, levels of CSPs identified from our cell-surface proteome do not track RNA levels from Purkinje cells obtained from either study (Buchholz et al., 2020; Kozareva et al., 2021). This discrepancy could be due to a combination of following: (1) transcriptomes and proteomes from the same cells often show at best modest correlations due to diverse post-transcriptional regulatory mechanisms including differential mRNA transport and stability, translation efficiency, and protein stability; (2) cell-type-specific RNA-seq data is typically captured from cell bodies or nuclei (although TRAP can capture translating mRNAs from dendrites), whereas our cell-surface proteomes are captured from the surfaces of Purkinje cells, whose dendritic surface area is two orders of magnitude larger than their somatic surface area; (3) our cell-surface proteomes also include proteins produced by neighboring cell types (see panels B–D below). The comparison in this panel suggests examples of each of the above possibilities. For example, RNAs for: (1) *Pcdh1* (#43), *Mxra8* (#47), and *Vcam1* (#51) are detected in neither TRAP nor snRNAseq datasets, suggesting higher protein stability relative to RNA stability; (2) *Omg* (#5), *Elfn2* (#41), and *Cldn11* (#46) are not detected in snRNAseq data but are present in TRAP data, suggesting dendritic local translation; (3) *Mertk* (#28) and *Cacna2d1* (#33) are not detected in Purkinje cells via TRAP or snRNAseq but are expressed highly in neighboring Bergmann glia (*Mertk*) and granule cells (*Cacna2d1*), suggesting proteomic detection of CSPs produced by neighboring cells.
(B) Schematic illustration of cell types in close contact with Purkinje cells in the cerebellar cortex. Magnified, a schematic of a Bergmann glia process in close contact with a Purkinje cell dendritic spine and a presynaptic terminal from a parallel fiber (a granule cell axon). iPEEL labels proteins produced not only by cells expressing HRP, but also by cells in close contact with HRP-expressing cells.
(C–E) In situ hybridization (ISH) of cell type marker genes *Mertk* (C) and *Cbln1* (D) encoding detected CSPs expressed in adult Bergmann glia and granule cells, respectively. As a control, *Armh4* is expressed in Purkinje cells (E; see also Figure S5C). Data are from the Allen Mouse Brain Atlas (Lein et al., 2007), http://mouse.brain-map.org/experiment/show/69752185 (C), http://mouse.brain-map.org/experiment/show/100145395 (D), http://mouse.brain-map.org/experiment/show/68080685 (E). ML, molecular layer; PCL, Purkinje cell layer; GrCL, granule cell layer; WML, white matter layer. Scale bar, 500 µm.
(F) Estimated coverage of iPEEL-derived mature Purkinje cell surface proteome. First, we generated a putative Purkinje cell CSP list, including proteins that contain a signal peptide according to UniProt annotation and are detected as expressed by Purkinje cells by snRNAseq (Kozareva et al., 2021). Since there is no definitive cutoff for gene expression in RNAseq data, we utilized multiple cutoff parameters: different levels of transcript counts per million reads [$\log_2$(counts per million +1) > 6, 7, or 8] and detection in varying percentages (50%, 60%, or 70%) of Purkinje cell nuclei. Then, we calculated the percentage of putative Purkinje CSPs detected in the iPEEL-generated proteome. We note that this coverage estimate is imperfect because signal peptide-containing proteins include those residing in certain intracellular compartments (e.g., the secretory pathway) without cell-surface localization (false positives) but exclude type II transmembrane proteins (false negatives).

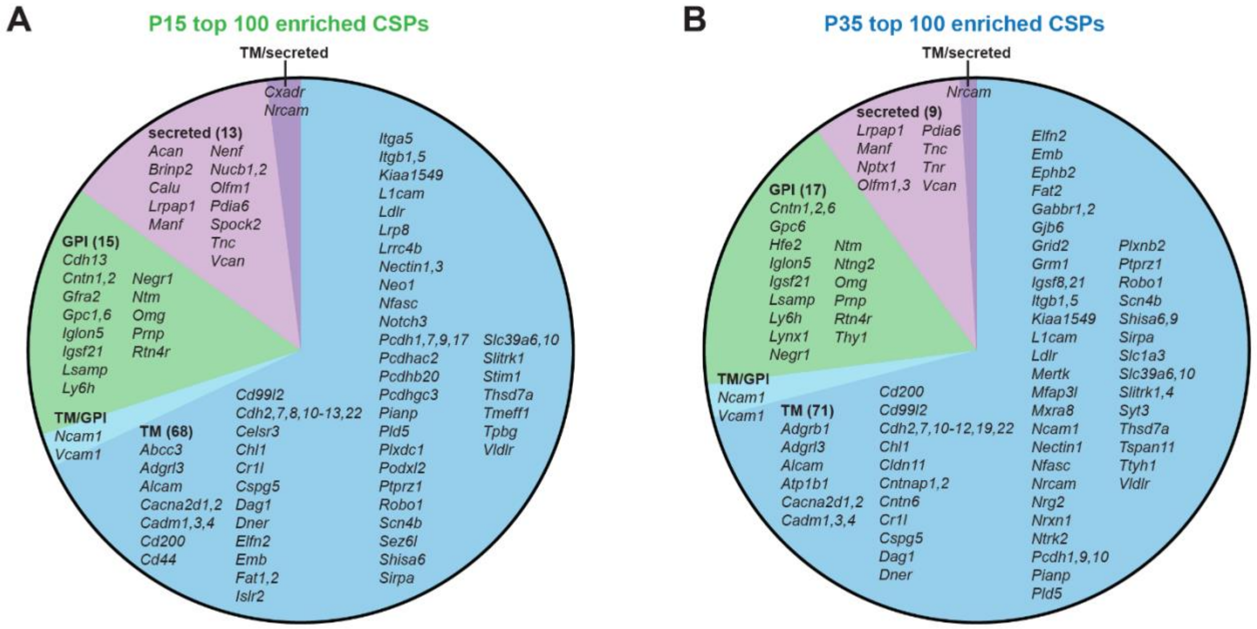

Figure S4. Molecular classes of most highly enriched CSPs of developing and mature Purkinje cells, related to Figure 4

Top 100 enriched CSPs of P15 (A) and P35 (B) Purkinje cells categorized by protein type (TM, transmembrane; GPI, glycosylphosphatidylinositol-anchored). Note that a few CSPs have alternative splicing isoforms that produce proteins of different molecular classes.

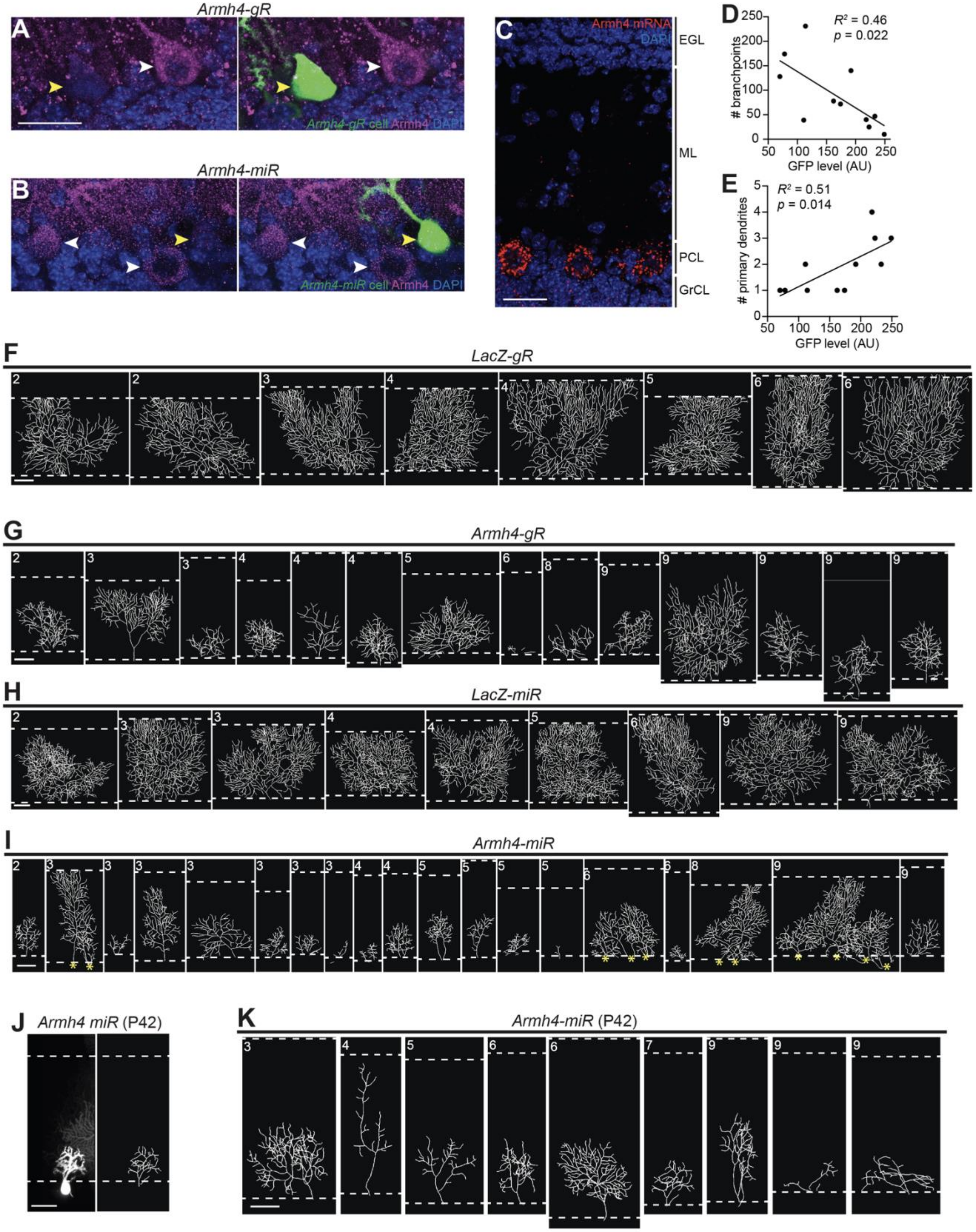

Figure S5. Validation of loss of Armh4 protein and morphological characteristics of *Armh4* loss-of-function Purkinje cells, related to Figures 5 and 6

(A and B) Immunostaining reveals loss of Armh4 protein in *Armh4-gR* (A) and *Armh4-miR* (B) cells. Green, *Armh4-gR/miR* cell expressing GFP; magenta, anti-Armh4 antibody staining; yellow arrowhead, loss of Armh4 immunostaining signal in *Armh4-gR/miR* cell; white arrowhead, Armh4 immunostaining signal in neighboring *wild-type* cell.
(C) RNAscope ISH of *Armh4* in P14 cerebellar cortex showing highly enriched *Armh4* expression in Purkinje cells. Blue, DAPI stain; red, *Armh4* ISH signal. EGL, external germinal layer; ML, molecular layer; PCL, Purkinje cell layer; GrCL, granule cell layer.
(D and E) Correlations between GFP levels (a proxy for miR levels) and the number of dendritic branchpoints (D) and primary dendrites (E) from images with fields-of-view in which multiple *Armh4-miR* Purkinje cells were present. AU, arbitrary unit.
(F–I) Dendritic arbor traces of *LacZ-gR* (F), *Armh4-gR* (G), *LacZ-miR* (H), and *Armh4-miR* (I) P21 Purkinje cells. Dotted white lines demarcate the molecular layer. The first and third cells from the left in (I) were imaged in fields of view excluding the top of the molecular layer; these cells did not contribute to height deficiency quantifications. Asterisks (*) denote the positions of cell bodies in images containing multiple *Armh4-miR* cells. Numbers on the top left indicate the cerebellar lobules from which cells originate.
(J) Representative image of P42 *Armh4-miR* Purkinje cell (left) and its dendritic arbor trace (right).
(K) Dendritic arbor traces of *Armh4-miR* P42 cells. Dotted white lines demarcate the molecular layer. Numbers on the top left indicate the cerebellar lobules from which cells originate.
Scale bars, 30 µm.

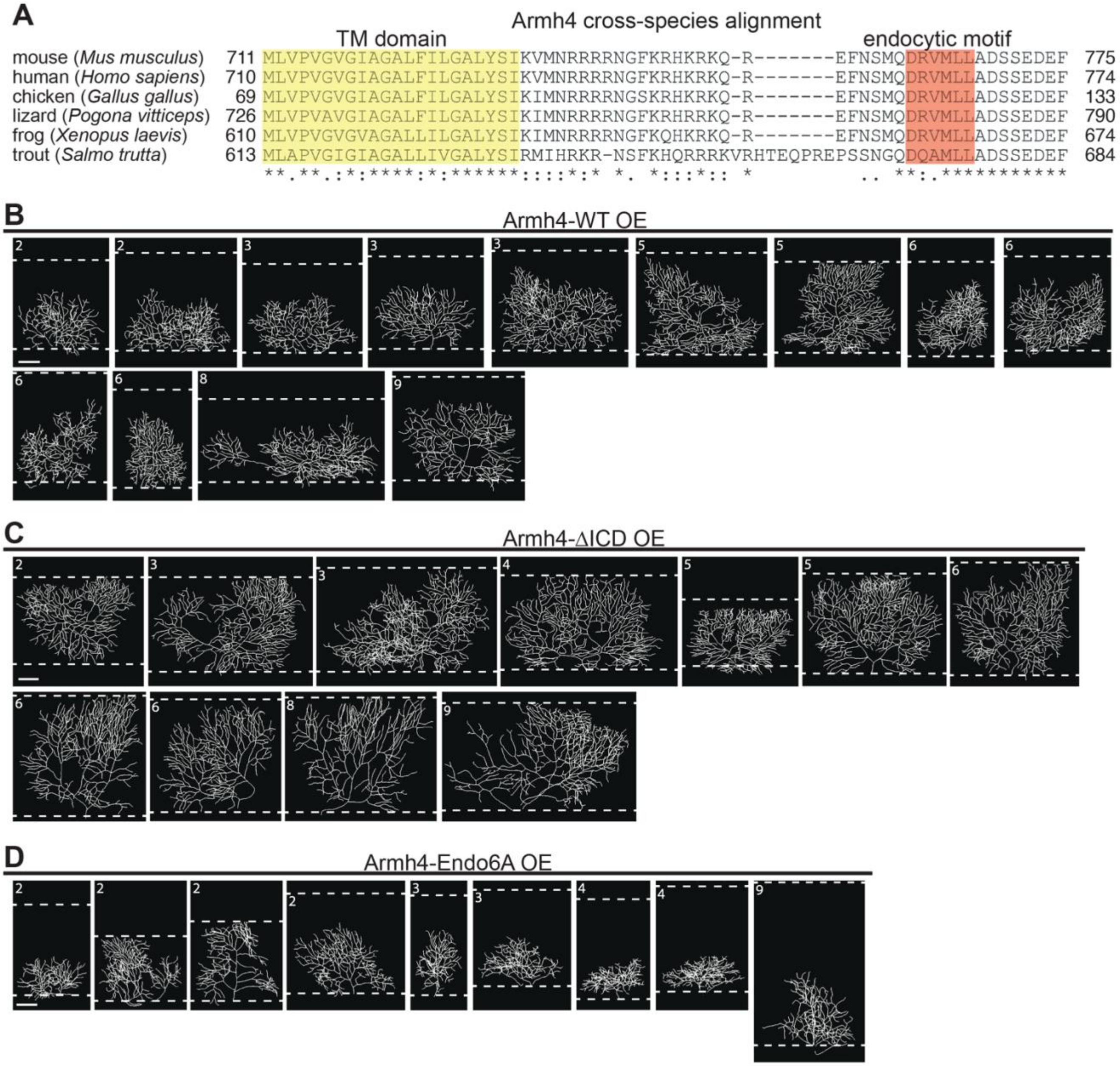

Figure S6. Sequence comparison of Armh4 across vertebrates and morphological characteristics of P21 Armh4 variant overexpression Purkinje cells, related to Figure 6

(A) Amino acid sequence alignment of the Armh4 transmembrane (TM, highlighted in yellow) and intracellular domains, including endocytic motif (highlighted in orange), over a wide range of vertebrates. Numbers represent amino acids in proteins starting from the start codon.

(B–D) Dendritic arbor traces of Armh4^{WT} (B), Armh4$^{\Delta ICD}$ (C), and Armh4^{Endo6A} (D) overexpression (OE) P21 Purkinje cells. Dotted white lines demarcate the molecular layer. Numbers on the top left indicate the cerebellar lobules from which cells originate. Scale bars, 30 µm.

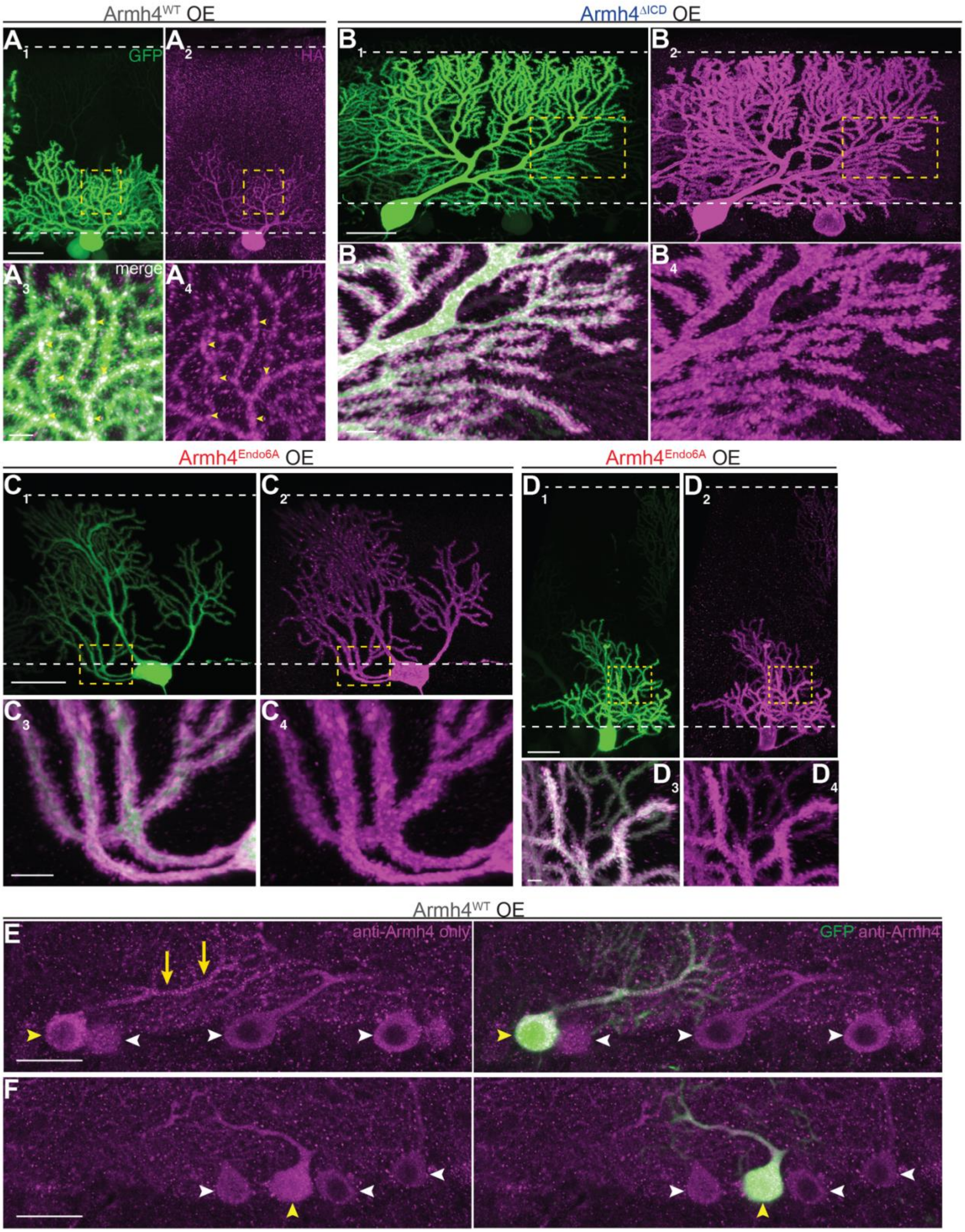

Figure S7. Subcellular localization of Armh4 variants overexpressed in Purkinje cells, related to Figure 6

(A–D) Additional confocal images of Purkinje cells sparsely overexpressing GFP and HA-tagged Armh4^{WT} (A), Armh4$^{\Delta ICD}$ (B), or Armh4^{Endo6A} (C and D). Top, images of whole dendritic arbors; bottom,

magnified images (from dashed yellow boxes) showing subcellular localization of overexpressed Armh4 variants; dashed white lines, top and bottom of cerebellar molecular layer. Arrowheads, intracellular Armh4^{WT}-HA puncta ($A_{3,4}$). Green, GFP; magenta, HA. Scale bar, 5 µm for insets ($A_{3,4}$–$D_{3,4}$); 30 µm for ($A_{1,2}$–$D_{1,2}$).

(E and F) Single confocal plane images of Purkinje cells sparsely overexpressing GFP and Armh4^{WT} (yellow arrowheads) confirm—by anti-Armh4 antibody staining—overexpression of Armh4 relative to neighboring Purkinje cells (white arrowheads). In Armh4 OE Purkinje cells, dendritic trunks are more brightly demarcated, suggesting more cell-surface expression; there are also dense intracellular puncta in dendrites (yellow arrows). Green, GFP; magenta, anti-Armh4 immunostaining signal. Scale bars, 30 µm.

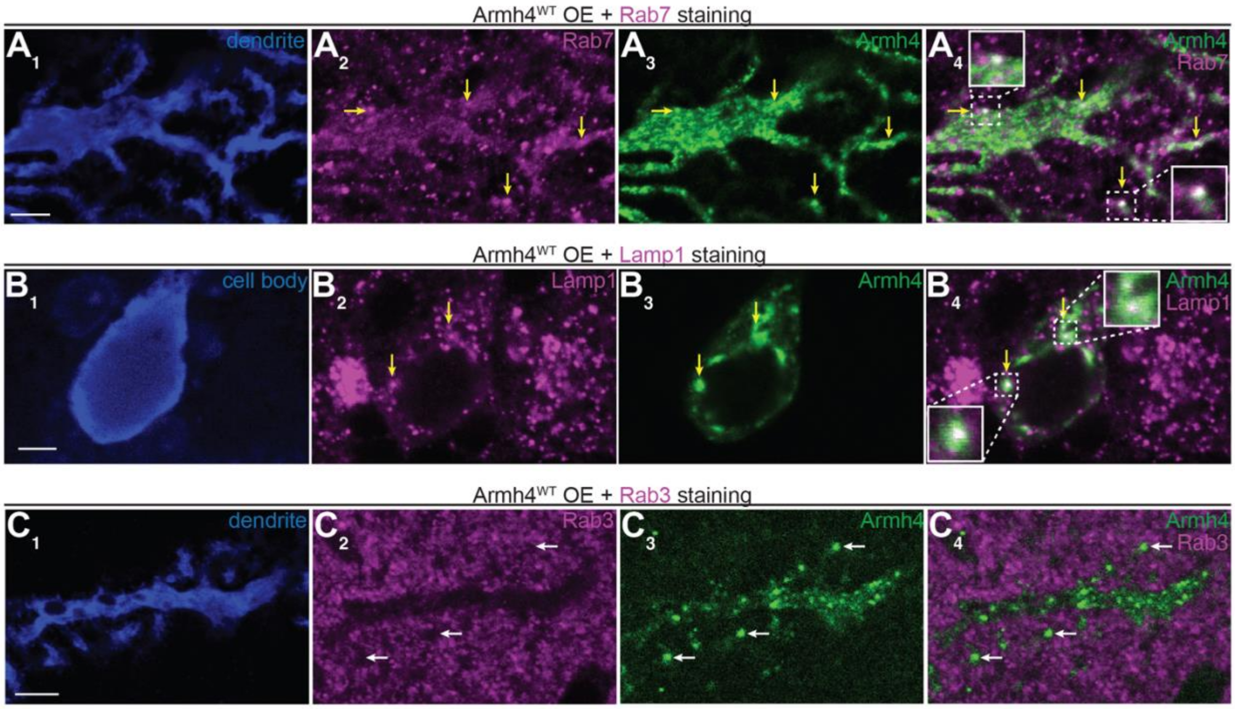

Figure S8. Colocalization of Armh4 with endolysosomal marker proteins, related to Figure 6
Immunostaining reveals partial colocalization of Armh4^{WT} with Rab7 and Lamp1 but not Rab3. Cell body and dendrites (blue, A_1–C_1), marker protein (magenta, $A_{2,4}$–$C_{2,4}$), Armh4 (green, $A_{3,4}$–$C_{3,4}$, by HA/V5 staining). We used N- or C-terminal HA- and V5-tagged Armh4^{WT} for these colocalization studies (STAR★Methods). We did not detect any difference between N- and C-terminal tags in Armh4 localization or dendrite morphogenesis phenotypes.

(A) Yellow arrows show co-localization between Armh4^{WT}-HA and Rab7, a marker of late endosomes. Inset, high magnification of co-localized Armh4^{WT}-HA and Rab7 puncta.

(B) Yellow arrows show co-localization between HA-Armh4^{WT} and Lamp1, a marker of lysosomes. Inset, high magnification of co-localized HA-Armh4^{WT} and Lamp1 puncta.

(C) White arrows show a lack of co-localization between Armh4^{WT}-V5 and Rab3, a marker of presynaptic terminals used as a negative control, which does not overlap with the labeled dendritic segments.

Scale bars, 5 μm.

Table S2. In vivo loss-of-function screen of proteome-informed candidate genes in Purkinje cell dendrite morphogenesis, related to Figure 5

candidate	method	phenotype	LOF reagent
Armh4	**gR pair**	**see Figures 5 and S5**	GAGCACTACCACCAAGTATT
			GCTCCAATGGTACTATCTGA
	**miR**	**see Figures 5 and S5**	TATGAGCAGACCAACTCTGAT
Cd47	gR pair	none observed	CCACATTACGGACGATGCAA
			GGCCTCATTGTAATCTCTAC
	MADM[1]	none observed	KO mouse, Jackson laboratory 003173
Cxadr	gR pair	none observed	ACGAGTAACGATGTCAAGTC
			CACTATCTGGTTATCAGACG
D430041D05Rik	miR	none observed	TCCAGTGAACCGACAGAGATA
Il6st	miR	**short arbors, supernumerary primary dendrites (2 – 6), migration defects**	AGGTCACTGTCATCAACAGAA
Islr2	gR pair 1	none observed	AGTTACGAAGGTCGCTCCAA
			AAGATTACGGTACTAAGGCG
	gR pair 2	none observed	ACGCACGCTGGGGGGTGCGC
			ATGTTACATTGCGTCGCCGA
	miR 1	**height deficiency**	AGTTTGCAGACTGTGCCTACA
	miR 2	**supernumerary primary dendrites (2 – 4), sometimes height deficiency**	AGTGCTGAGTCAGCTCAAGAA
Ntm	gR pair	**supernumerary primary dendrites (2 – 6), branching pattern**	TGACAACCGAGTCACCCGGG
			ACCTTCACTCTTCGTACCAC
	miR 1	none observed	CTACAGTAACCTGGAGACATA
	miR 2	none observed	ACCAGTGGTACGAAGAGTGAA
Pianp	gR pair	none observed	GGTCCCAAGATCACGTCGGC
			GACCCCAACTCGGTCAATCC
Podxl2	gR pair	none observed	AACACCGTGGGACTCTACTC
			GTGGCGTCCATGGAAGACCC
	miR	**migration defects**	CAGCTGAAGCTCACTCACTAA
Spock2	gR pair	none observed	GTTGCAAGGACTCCATCGGT
			CAGCCAAGGCGACCACGGCG
Thsd7a	gR pair	**height deficiency**	TGTTTAAGCACGTCACGTCC
			GGCACAAGGAATTGTACGAC
	MADM[1]	none observed	KO mouse, KOMP/IMPC[2]
Tmeff1	gR pair 1	none observed	ATTGTTATAGGAACTCCCGT
			GTACAAGGCCGAGTGTGACG
	gR pair 2	none observed	GTGTGGACCGTGCAAGTACA
			AACGGCGTTTACCTGTGCAG
	gR pair 3	none observed	GTACTTGCACGGTCCACACT
			GCACCAGAAAGACATAACCG
	miR	**cell health phenotype (e.g. blebbing) in a few cells**	GAAGATGGAGATGGTTTGAAA
Tmeff2	miR 1	none observed	CCTCCTTAAGTGACTGCCAAA
	miR 2	none observed	ACAGCAGAGTGAGATACTTGT

[1]MADM (mosaic analysis with double markers) is a method for analyzing sparse homozygous mutant cells in a heterozygous background (Zong et al., 2005); we utilized mice from a collection of MADM alleles (Contreras et al., 2021).

[2]*Thsd7a* KO mice were a gift from S.B. Nelson (Clark et al., 2020) and were generated by the Knockout Mouse Project (KOMP) of the International Mouse Phenotyping Consortium (IMPC).
